# The transcription factor DUX4 orchestrates translational reprogramming by broadly suppressing translation efficiency and promoting expression of DUX4-induced mRNAs

**DOI:** 10.1371/journal.pbio.3002317

**Published:** 2023-09-25

**Authors:** Danielle C. Hamm, Ellen M. Paatela, Sean R. Bennett, Chao-Jen Wong, Amy E. Campbell, Cynthia L. Wladyka, Andrew A. Smith, Sujatha Jagannathan, Andrew C. Hsieh, Stephen J. Tapscott

**Affiliations:** 1 Human Biology Division, Fred Hutchinson Cancer Center, Seattle, Washington State, United States of America; 2 Molecular and Cellular Biology Program, University of Washington, Seattle, Washington State, United States of America; 3 Department of Biochemistry and Molecular Genetics, University of Colorado Anschutz Medical Campus, Aurora, Colorado, United States of America; 4 RNA Bioscience Initiative, University of Colorado Anschutz Medical Campus, Aurora, Colorado, United States of America; 5 Departments of Medicine and Genome Sciences, University of Washington, Seattle, Washington State, United States of America; 6 Department of Neurology, University of Washington, Seattle, Washington State, United States of America; 7 Clinical Research Division, Fred Hutchinson Cancer Center, Seattle, Washington State, United States of America; Johns Hopkins University, UNITED STATES

## Abstract

Translational control is critical for cell fate transitions during development, lineage specification, and tumorigenesis. Here, we show that the transcription factor double homeobox protein 4 (DUX4), and its previously characterized transcriptional program, broadly regulates translation to change the cellular proteome. DUX4 is a key regulator of zygotic genome activation in human embryos, whereas misexpression of DUX4 causes facioscapulohumeral muscular dystrophy (FSHD) and is associated with MHC-I suppression and immune evasion in cancer. We report that translation initiation and elongation factors are disrupted downstream of DUX4 expression in human myoblasts. Genome-wide translation profiling identified mRNAs susceptible to DUX4-induced translation inhibition, including those encoding antigen presentation factors and muscle lineage proteins, while DUX4-induced mRNAs were robustly translated. Endogenous expression of DUX4 in human FSHD myotubes and cancer cell lines also correlated with reduced protein synthesis and MHC-I presentation. Our findings reveal that DUX4 orchestrates cell state conversion by suppressing the cellular proteome while maintaining translation of DUX4-induced mRNAs to promote an early developmental program.

## Abstract 2

Cellular reprogramming requires multifaceted regulation of gene expression. This study shows that DUX4 modulates multiple translational regulators to broadly suppress protein synthesis while promoting expression of DUX4-induced genes, suggesting a natural role in cell state conversion during development and a pathological role when expressed in cancer or muscular dystrophy.

## Introduction

The *double homeobox protein 4* (*DUX4*) gene encodes a transcription factor that is expressed in immune-privileged niches such as the preimplantation embryo [1,2], testis [3], and, possibly, thymus [4]. DUX4 is briefly expressed in the 4-cell human embryo and serves as a key transcriptional activator of the zygotic genome, driving expression of hundreds of coding genes and repetitive retroelements [1,2]. In addition to zygotic genome activation (ZGA), regulation of mRNA degradation and translation is essential to rapidly diversify the proteome during early development [5] and has been associated with increased developmental potential of human preimplantation embryos [6]. It is becoming abundantly clear that translational control, both globally and at the level of individual transcripts, helps mediate cell fate transitions. This includes the shift from the maternal to the embryonic developmental program, the balance of stem cell self-renewal and differentiation, and the plasticity of cancer [7,8].

Emerging evidence has shown that broad translational suppression is a hallmark of reprogramming in embryonic and somatic stem cells [9–11], where low rates of translation are thought to promote an undifferentiated state. This is further supported by the finding that a rare population of totipotent mouse embryonic stem cells (ESCs) known as 2-cell-like cells (2CLCs), thought to recapitulate the naïve state of the preimplantation embryo [12], exhibit global repression of nascent protein synthesis [13,14]. Mouse Dux and human DUX4 belong to the conserved DUXC family of proteins found in eutherian mammals [15]. Functionally, expression of mouse Dux reprograms these rare populations of 2CLCs to have expanded developmental potential [1,2,12], and human DUX4 has been reported to drive a similar totipotent program in human induced pluripotent stem cells (iPSCs) and ESCs [2,16].

*DUX4* is also the causative gene of facioscapulohumeral muscular dystrophy (FSHD), a complex genetic disorder that results in epigenetic derepression of the *DUX4* locus in skeletal muscle and progressive muscle atrophy [17–20]. The aberrant expression of DUX4 in skeletal muscle activates expression of genes associated with germline and stem cell development [21,22], characteristic of the early embryonic ZGA program. Although DUX4 is sporadically expressed in approximately 0.1% of FSHD muscle cells in culture [3,23], DUX4 target gene activation results in a host of pathogenic features including impaired myogenesis [24], oxidative stress and DNA damage [25,26], compromised mRNA quality control [27,28], and inflammation [29,30].

In addition to a role in FSHD, recent analysis of nearly 10,000 cancer transcriptomes from 33 different cancer types revealed *DUX4* to be one of the most commonly expressed cancer-associated genes [31]. Full-length *DUX4* expression in diverse cancer types is strongly correlated with increased expression of high-confidence DUX4 targets activated in the embryo [31]. DUX4 expression in cancers was associated with decreased major histocompatibility complex class I (MHC-I) expression, resistance to checkpoint inhibitors, and decreased patient survival rates [31]. DUX4 expression in several cancer cell lines was sufficient to prevent the induction of MHC-I expression in response to interferon gamma (IFNγ); however, it was unclear whether this represented an activity restricted to MHC-I or a broader activity of DUX4 in regulating protein expression.

In this study, we have implicated the DUX4 transcriptional program as a driver of broad translational suppression that reprograms de novo protein synthesis. We initially focused on the DUX4 suppression of MHC-class I and related interferon-stimulated proteins to characterize the mechanisms of protein suppression. We found that a brief pulse of DUX4 expression disrupts several key regulators of translation initiation and elongation, which are sufficient to suppress protein synthesis of IFNγ-stimulated MHC-I and immunoproteasome (iProteasome) subunits. Moreover, high-throughput translational profiling with ribosome footprinting and polysome gradients showed significant changes in MHC-I translational efficiency (TE) and broad translational suppression of many cellular mRNAs, whereas DUX4-induced mRNAs were robustly translated. Taken together, DUX4 activation of its transcriptional program resulted in the replacement of the prior cellular proteome and lineage identity with the DUX4-induced proteome enriched for ZGA-associated proteins. We propose that coordinated regulation of transcription and translation is employed by DUX4 to reshape the cellular translatome in both development and disease.

## Results

### DUX4 activity induces prolonged suppression of antigen presentation factors

We recently reported that DUX4 blocks IFNγ-stimulated induction of MHC-I and surface antigen presentation [31]. To determine the mechanism of DUX4-induced MHC-I regulation, we used a well-characterized cellular model system of human myoblasts with a doxycycline (DOX)-inducible *DUX4* transgene (MB135iDUX4) [32]. DUX4 expression occurs in transient bursts in rare populations of ESCs [2,16] and is sporadically misexpressed in FSHD muscle cells [3,23], making it difficult to characterize downstream mechanisms endogenously. We have previously demonstrated that a short “pulse” of DUX4 in MB135iDUX4 myoblasts induced a transcriptional program representative of FSHD muscle cells and the early cleavage-stage embryo [33]. Pulsed DUX4 expression in this cell culture system enabled reproducible and synchronized DUX4 induction, permitting the investigation of mechanisms downstream of DUX4 that may have otherwise been masked by heterogeneous populations of DUX4-expressing cells.

**Fig 1:**
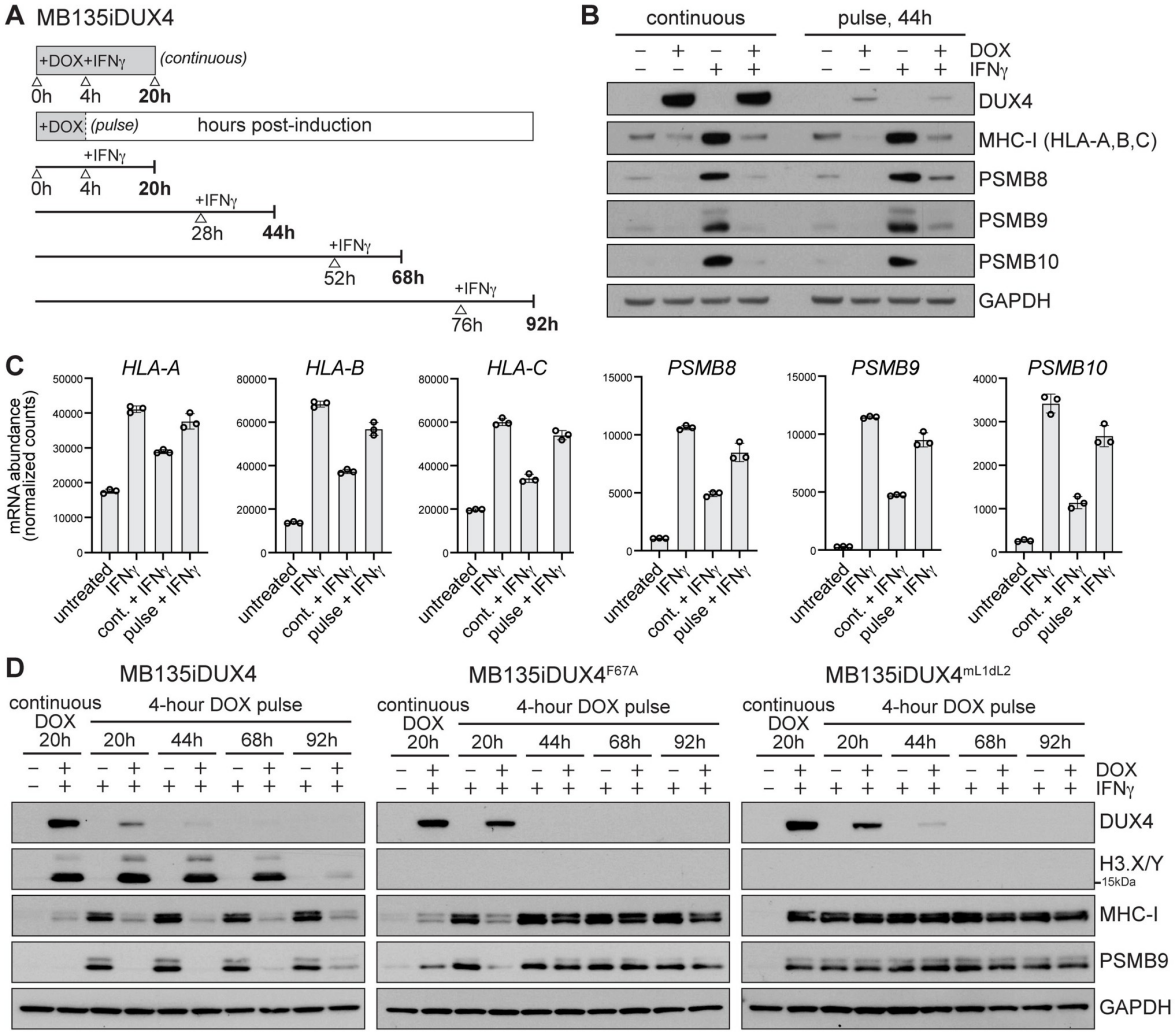
Brief expression of DUX4 results in long-term suppression of MHC-I and iProteasome subunits. (**A**) Schematic of experimental time course. (**B**) Immunoblot analysis following treatment with or without DOX and IFNγ as noted. Cells expressing DUX4 continuously (left) or a pulse of DUX4 (right). (**C**) Normalized RNA-seq read counts; data represent mean ± SD of biological replicates, *n =* 3. Source data available in S1 Data. (**D**) Immunoblot analysis of extended experimental time course outlined in (**A**). DOX-inducible MB135 myoblasts expressing active DUX4 (left) versus transcriptionally inactive DUX4 carrying F67A mutation in the DUX4 DNA binding domain (center), or mutation of the first (L)LxxL(L) motif and deletion of the second (L)LxxL(L) motif within the C-terminal activation domain (mL1dL2, right; see [34] for mutation sequences). DUX4 E14-3 antibody detects epitope in active DUX4 and mutant DUX4 proteins. DUX4 targets H3.X and H3.Y are expressed upon induction of active DUX4, but not DUX4(F67A) or DUX4(mL1dL2). GAPDH serves as loading control. DOX, doxycycline; DUX4, double homeobox protein 4; IFNγ, interferon gamma; MHC-I, major histocompatibility complex class I.

Using our MB135iDUX4 cell culture system, we tested both the immediate effect of continuous DUX4 expression, as well as the prolonged consequences following a brief pulse of DUX4 on the expression levels of several IFNγ-induced factors involved in immunogenic antigen presentation, including canonical MHC-I subunits (HLA-A, HLA-B, HLA-C) and iProteasome subunits (PSMB8, PSMB9, PSMB10). MB135iDUX4 myoblasts were treated with DOX for 20 hours or for a 4-hour period followed by washout to induce a “continuous” versus a “pulse” of DUX4 expression, respectively. Cells were exposed to IFNγ for the final 16 hours prior to harvest, collecting terminal time points at 20 hours for the continuous treatment, and at 44 hours for the pulse (40 hours after the DOX washout) (Fig 1A). Cells stimulated with IFNγ showed elevated protein levels of MHC-I and iProteasome subunits, whereas IFNγ induction of MHC-I and the iProteasome was suppressed in myoblasts continually expressing DUX4 (Fig 1B, left). Remarkably, a pulse of DUX4 elicited the same degree of suppression despite having diminished levels of DUX4 protein (Fig 1B, right).

In a recent study, we found that the DUX4 protein was sufficient to inhibit IFNγ induction of interferon-stimulated genes (ISGs) at the mRNA level by interacting with STAT1 and preventing RNA Pol-II recruitment to STAT1-regulated genes [34]. Our current findings that suppression of IFNγ-stimulated factors persisted following a transient pulse of DUX4 suggested an additional mechanism of regulation downstream of DUX4 protein expression. We compared mRNA levels of MHC-I and iProteasome subunits following continuous or pulsed DUX4 expression and found that transcriptional suppression of IFNγ signaling by continuous DUX4 mostly recovered at 44 hours following the pulse of DUX4 (Fig 1C). These data indicated that a pulse of DUX4 induced posttranscriptional suppression of MHC-I and iProteasome proteins through a method distinct from its interaction with STAT1 to suppress ISG mRNA induction.

To determine the necessity of DUX4 transcriptional activity for long-term protein suppression of MHC-I and the iProteasome, we performed a time course with active DUX4 and transcriptionally inactive DUX4 mutants carrying either a mutation in the DUX4 DNA binding domain (F67A) or mutations within the (L)LxxL(L) motifs of the C-terminal activation domain (mL1dL2). A prolonged time course revealed that protein suppression persisted for several days following a pulse of active DUX4, whereas transcriptionally inactive DUX4 mutants were insufficient to suppress MHC-I and PSMB9 at later time points (Fig 1D). Suppression of MHC-I and PSMB9 induction at the 20-hour time point was observed with the DUX4 F67A mutant, but not the mL1dL2 mutant (Fig 1D). This is likely mediated by inhibition of interferon signaling and ISG transcription by the DUX4 protein that requires the C-terminal activation domain and (L)LxxL(L) motifs [34], whereas long-term suppression of MHC-I and PSMB9 that persists after the loss of DUX4 protein required the transcriptional activity of DUX4. Collectively, these data suggest that DUX4 acts as a repressor of antigen presentation factors through 2 distinct mechanisms. Here, we focus on the finding that transient DUX4 expression activates a transcriptional program required for prolonged protein suppression.

Subcellular fractionation of mRNAs and proteins showed that a pulse of DUX4 did not disrupt *HLA-A*, *HLA-B*, *HLA-C*, *PSMB8*, *PSMB9*, or *PSMB10* mRNA nuclear export or protein localization to the cytoplasm following IFNγ simulation (S1 Fig). Additionally, treatment with proteasome inhibitor MG132 or autophagy inhibitor Bafilomycin did not rescue suppression of IFNγ-stimulated MHC-I, PSMB8, PSMB9, or PSMB10 following a pulse of DUX4 (S2 Fig), eliminating protein degradation as a causal mechanism. iProteasome production of immunogenic antigens has also been linked to MHC-I protein stability; however, siRNA-mediated knockdown of iProteasome catalytic subunits PSMB8 and PSMB9 in parental MB135 myoblasts did not impact IFNγ-induced MHC-I levels (S3A Fig). Furthermore, treatment with ONX-0914, a selective inhibitor of the iProteasome, did not reduce IFNγ-induced MHC-I levels (S3B Fig). Thus, MHC-I stability does not require iProteasome-dependent proteolysis. Together, these data suggest that DUX4 posttranscriptionally suppresses MHC-I and the iProteasome independently through methods of translational inhibition.

### DUX4 modulates multiple pathways involved in translational regulation

**Fig 2:**
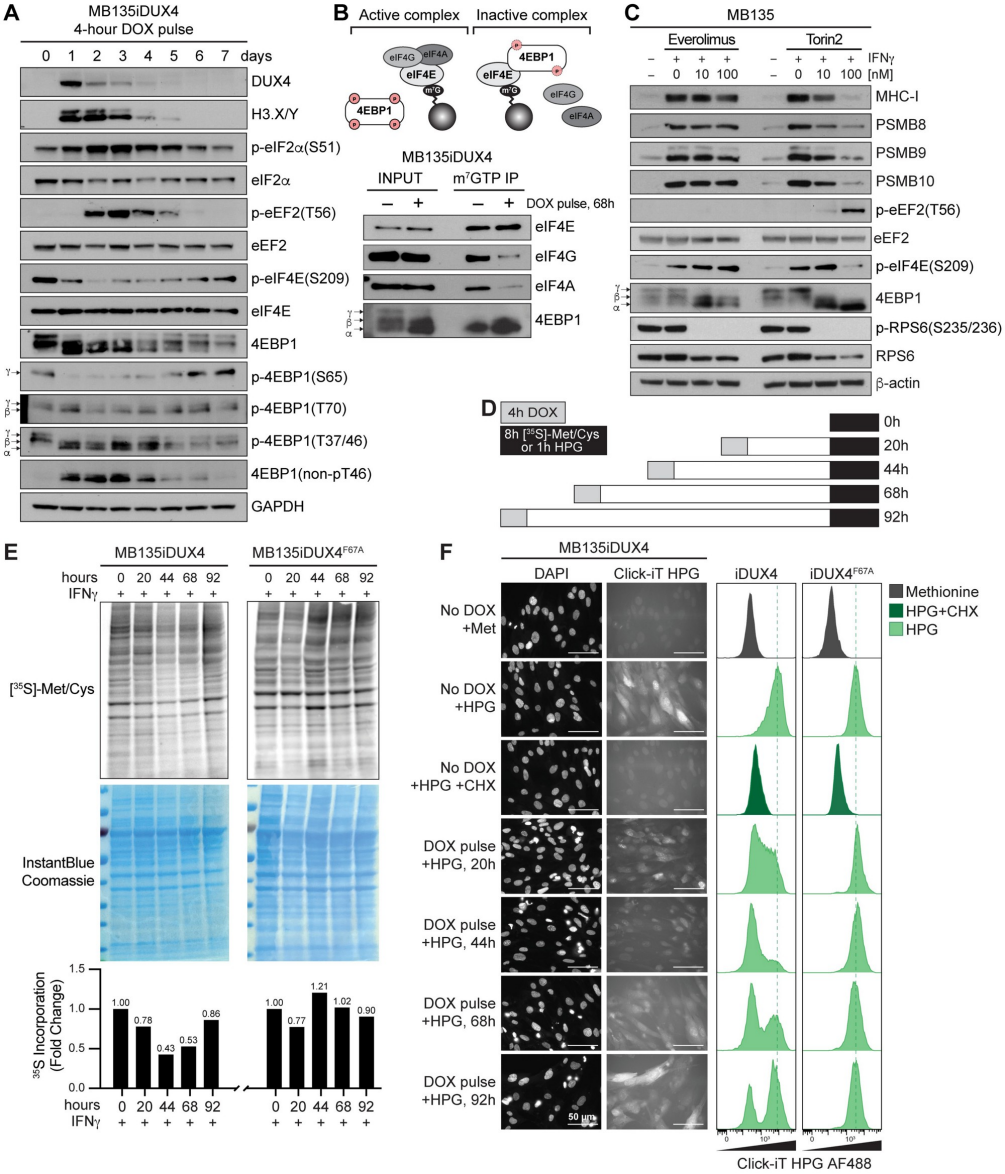
A pulse of DUX4 negatively regulates the status of multiple translational regulators and broadly suppresses nascent protein synthesis. (**A**) Immunoblot analysis 0–7 days following a 4-hour pulse of DOX in MB135iDUX4 myoblasts. Alpha, beta, and gamma correspond to the phosphorylated forms of 4EBP1. GAPDH serves as loading control. (**B**) Schematic of cap-dependent translation initiation complex (top) and immunoblot analysis of m^7^GTP pull-downs (bottom). (**C**) Immunoblot analysis of MB135 myoblasts treated with mTORC1 inhibitors Everolimus or Torin2. (**D**) Schematic of experimental time course for metabolic labeling with 35S or HPG. (**E**) Autoradiograph of samples pulsed with active DUX4 (top, left) or DNA-binding mutant F67A (top, right); Coomassie stain of total protein (middle); quantification of relative 35S signal normalized to paired 0-hour condition (bottom). Source data available in S1 Data. (**F**) Immunofluorescence (left) and flow cytometry (right) of HPG/Click-iT labeled proteins (scale bars, 50 μm). DOX, doxycycline; DUX4, double homeobox protein 4; HPG, L-homopropargylglycine; mTORC1, mechanistic target of rapamycin complex 1; m^7^GTP, 7-methylguaniosine 5′-triphosphate; 4EBP1, 4E-binding protein 1.

We previously reported that DUX4 induces nuclear double-stranded RNA (dsRNA) accumulation resulting in phosphorylation of PKR and the eukaryotic initiation factor eIF2-alpha [35]. In a time-course experiment, phosphorylation of eIF2-alpha persisted for several days following a pulse of DUX4 (Fig 2A). PKR plays a key role in blocking translation through the phosphorylation of eIF2-alpha at serine 51, which results in destabilized eIF2-GTP/Met-tRNAi ternary complexes [36]. We generated a *PKR* knockout (KO) in MB135iDUX4 myoblasts and observed no differences in DUX4 suppression of MHC-I or iProteasome subunits between wild-type (WT) and PKR KO cells with either continuous or pulsed DUX4 induction despite the rescue of DUX4-induced eIF2-alpha phosphorylation (S3C–S3E Fig). These results demonstrated that inhibition of eIF2-alpha was not necessary for DUX4-mediated protein suppression of antigen presentation factors.

Notably, a pulse of DUX4 in MB135iDUX4 myoblasts also caused prolonged dephosphorylation of 4E-binding protein 1 (4EBP1) (Fig 2A), a negative regulator of cap-dependent translation. In its hypophosphorylated state (denoted as alpha and beta), 4EBP1 functions as a scaffold protein that sequesters eukaryotic initiation factor 4E (eIF4E), a major mRNA cap-binding protein, to prevent engagement in translation initiation. Active eIF4E is marked by phosphorylation at serine 209 [37], and indeed, a pulse of DUX4 resulted in a loss of phospho-eIF4E (Fig 2A). Several protein kinases are assumed to phosphorylate 4EBP1, the best characterized being mechanistic target of rapamycin complex 1 (mTORC1). Therefore, we investigated other effectors downstream of mTORC1 signaling pathways known to regulate translation. We found that a pulse of DUX4 also increased phosphorylation of eukaryotic elongation factor 2 (eEF2) on threonine 56 (Fig 2A), a functional modification that disables eEF2 in mediating ribosome elongation during mRNA translation. Collectively, these data strongly suggest that DUX4 has adverse effects on both translation initiation and elongation.

Hypophosphorylated forms of 4EBP1 antagonize eIF4E by sequestering it from eIF4F protein complex formation with other translation initiation complex subunits eIF4A and eIF4G. Levels of active eIF4E are rate limiting for eIF4F complex assembly and cap-dependent translation initiation [38]. To directly measure if DUX4 disrupts the cap-binding capacity of the eIF4F complex, we conducted a cap pull-down assay by incubating sepharose beads coupled to 7-methylguaniosine 5′-triphosphate (m^7^GTP) with whole cell lysate extracted from MB135iDUX4 myoblasts 3 days after a DUX4 pulse. The m^7^GTP beads failed to coprecipitate eIF4G and eIF4A following a pulse of DUX4 compared to untreated cell lysate, despite equivalent precipitation of eIF4E. Instead, active complex subunits were displaced by inhibitory 4EBP1 as indicated by increased precipitation of hypophosphorylated 4EBP1 with eIF4E (Fig 2B). These data demonstrate that DUX4 disrupts the eIF4F complex, likely having substantial effects on cellular translation.

To evaluate the sufficiency of mTOR inactivation to block MHC-I and iProteasome protein levels in the absence of DUX4, we treated MB135 myoblasts with mTOR inhibitors everolimus or Torin2. While everolimus, a second-generation rapamycin analog, blocked S6K phosphorylation of RPS6, it did not efficiently target 4EBP1 hypophosphorylation or reduce eIF4E phosphorylation. Furthermore, treatment with everolimus followed by IFNγ did not inhibit MHC-I or iProteasome expression (Fig 2C, left; S4A Fig), indicating that the S6K branch of mTORC1 signaling is not sufficient on its own to suppress protein synthesis of MHC-I and the iProteasome subunits. Conversely, treatment with Torin2, an ATP-competitive inhibitor of mTOR, blocked phosphorylation of RPS6, resulted in robust phosphorylation of eEF2, hypophosphorylation of 4EBP1, and reduced eIF4E phosphorylation. Treatment with Torin2 suppressed MHC-I, PSMB8, PSMB9, and PSMB10 protein levels (Fig 2C, right) while having no significant effect on IFNγ induction of MHC-I and iProteasome subunits at the mRNA level (S4A Fig). These findings support previous observations the 4EBP-eIF4E axis, independent of RPS6, can significantly control cap-dependent translation [39], and demonstrated that combined eIF4E and eEF2 inactivation was sufficient to suppress antigen presentation factors. Additionally, MB135 cells treated with 4EGI-1, a small molecule that pharmacologically mimics 4EBP function and inhibits eIF4E [40], suppressed IFNγ-induced MHC-I and iProteasome subunits at the protein level with no significant effect on mRNA levels (S4B and S4C Fig). Together, these findings indicate that DUX4 expression alters the activity of several key regulators of translation initiation and elongation, which are sufficient to suppress protein expression of antigen presentation factors.

Response to cellular hypoxia, metabolic signaling, oxidative stress, and DNA damage are reported to be involved in early embryonic development [41–47] and contribute to the pathogenicity of DUX4 in FSHD [25,26,29,48,49]. These pathways have also been implicated in protein synthesis inhibition. Therefore, we investigated whether agents that activate each of these stress pathways would be sufficient to recapitulate the suppression of MHC-I and PSMB9 protein levels independent of DUX4. MB135 myoblasts treated with hydrogen peroxide to induce oxidative damage, DNA-damaging agent etoposide, or cobalt chloride to mimic hypoxic stress showed only slight suppression of IFNγ-induced MHC-I and PSMB9 (S5 Fig), much less than that observed following a pulse of DUX4. Therefore, it is likely that multiple pathways modulate protein suppression downstream of DUX4.

### Transient DUX4 activity broadly suppresses nascent protein synthesis

Uncoupling of the transcriptome and proteome downstream of DUX4 is not limited to antigen presentation factors. We previously reported a discordant relationship between RNA and protein levels for a multitude of mRNAs in DUX4-expressing cells [28]. This, in combination with the dysregulation of multiple key translational regulators (Fig 2A), suggested that DUX4 might broadly alter cellular translation. Indeed, metabolic labeling with 35S-methionine/cysteine in MB135iDUX4 myoblasts treated with IFNγ showed that de novo protein synthesis was transiently suppressed following a pulse of DUX4, with an approximate 50% reduction at the 68-hour time point (Fig 2D and 2E), whereas induction of transcriptionally inactive DUX4(F67A) did not alter nascent protein synthesis (Fig 2E, right).

We confirmed DUX4 inhibition of protein synthesis by labeling cells with methionine analog L-homopropargylglycine (HPG) followed by fixation and Click-iT chemistry, wherein fluorescence microscopy and flow cytometry showed a dramatic reduction in HPG-labeled peptides after a pulse of DUX4 (Fig 2F). As seen with 35S-methionine/cysteine labeling, HPG signal was lowest 44 hours after a pulse of DUX4, comparable to the degree of translational suppression induced by cycloheximide (CHX) treatment alone, and nascent protein synthesis started to recover in a subset of cells by 68 to 92 hours. These findings establish long-lived, yet transitory, suppression of protein synthesis downstream of the DUX4 transcriptional program that encompassed IFNγ-induced MHC-I and PSMB9 expression.

### Ribosome footprinting reveals a DUX4-induced loss of 5′ ribosome occupancy

**Fig 3:**
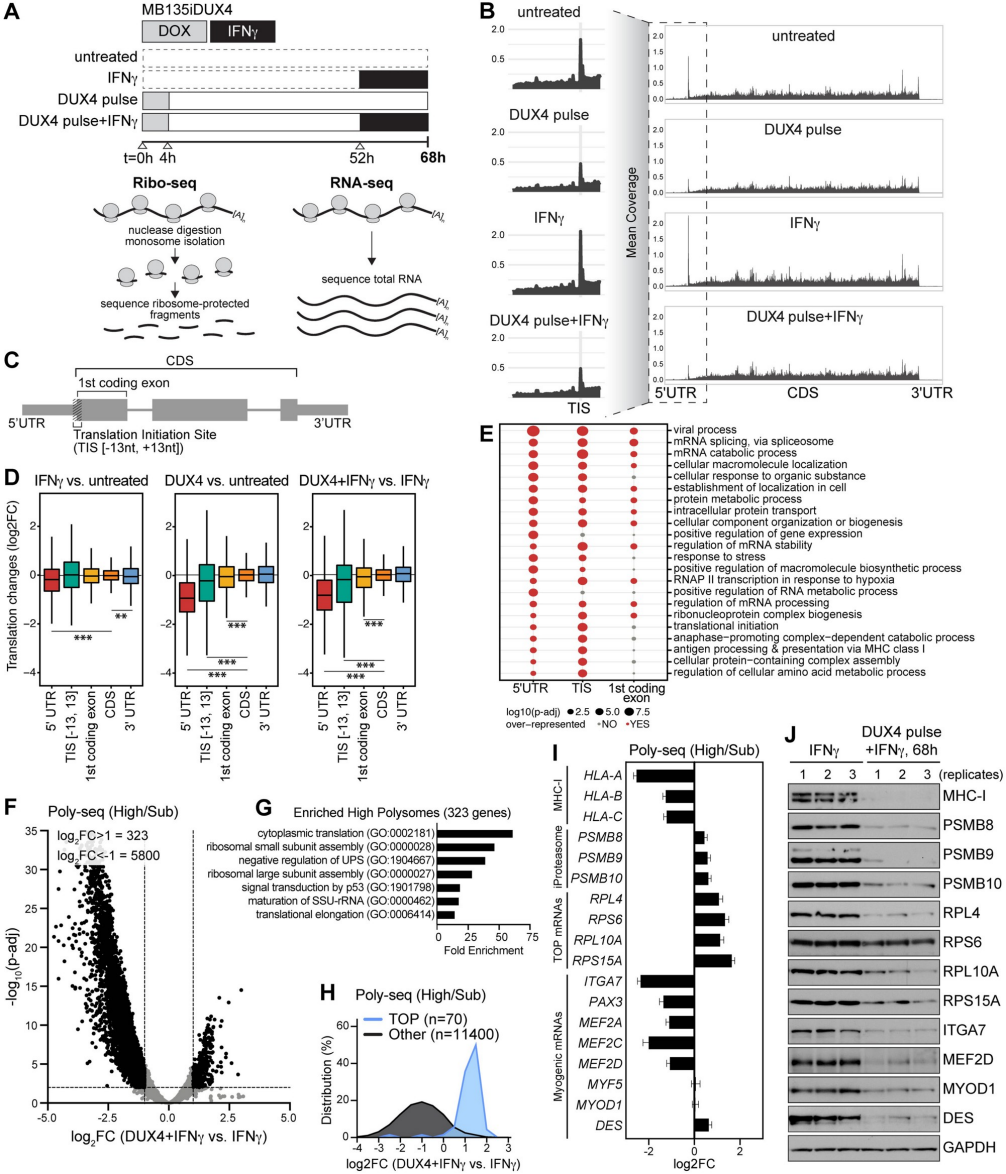
Ribosome footprinting and polysome profiling reveal DUX4 suppressive effects on translation efficiency. (**A**) Experimental schematic illustrating genome-wide quantification of mRNA counts and RPFs using RNA-seq and ribosome profiling (Ribo-seq). (**B**) Metagene analysis of RPFs; see S1 Data. (**C**) Schematic of annotated mRNA features 5′ UTR, TIS (−/+ 13 nt around start codon), first coding exon, CDS, and 3′ UTR. (**D**) Box plots of translational changes occurring at the level of mRNA features (|log2FC > 1|, p-adj < 0.05); see S7 Fig and S2 Data. Statistical comparisons were conducted using one-way ANOVA, * *p* < 0.001, ** *p* < 0.0001, *** *p* < 2.2 × 10^−16^. (**E**) Gene Ontology analysis for transcripts with significantly decreased ribosome occupancy in 5′ UTR, TIS, and first coding exon in MB135iDUX4 myoblasts treated with a DUX4 pulse+IFNγ versus IFNγ; see S3 Data. (**F**) Differential RNA-seq analysis of mRNA levels in high polysome fractions relative to sub-polysome fractions (high/sub). Volcano plot showing log2 fold-change differential polysome abundance in cells treated with DUX4 pulse+IFNγ harvested at 68 hours versus IFNγ (significance defined as basemean>50, |log2FC>1|, p-adj<0.01); see S4 Data. (**G**) Gene Ontology analysis for transcripts with significantly increased polysome abundance. (**H**) Relative polysome abundance for 5′ TOP mRNAs compared to all other mRNAs; see S4 Data. (**I**) Log2 fold-change in polysome abundance of MHC-I mRNAs, iProteasome subunits, select TOP mRNAs, and myogenic factors. Data represent mean ± SD of biological replicates, *n =* 3; see S4 Data. (**J**) Immunoblot analysis of total protein lysate from polysome profiling samples representing biological replicates, *n* = 3. GAPDH serves as loading control. CDS, coding sequence; DUX4, double homeobox protein 4; IFNγ, interferon gamma; MHC-I, major histocompatibility complex class I; nt, nucleotide; RNA-seq, RNA sequencing; RPF, ribosome-protected fragment; TIS, translation initiation site; TOP, terminal oligopyrimidine; UTR, untranslated region.

To globally profile translational regulation downstream of DUX4, we performed ribosome footprinting (Ribo-seq) paired with RNA sequencing (RNA-seq) on MB135iDUX4 myoblasts with 4 treatment conditions: untreated, IFNγ alone, DUX4 pulse harvested at 68 hours, and DUX4 pulse+IFNγ harvested at 68 hours (Fig 3A). Our sequencing reads representing ribosome-protected fragments (RPFs) displayed 3-nucleotide (nt) periodicity and exhibited lengths of 26 to 29 nt (S6 Fig). Metagene analysis showed the majority of RPFs mapped to the coding region (CDS) and revealed a profound depletion of Ribo-seq reads mapping to the mRNA region surrounding the translation initiation site (TIS) in samples pulsed with DUX4 (Fig 3B).

We calculated differential TE of steady-state mRNAs by measuring Ribo-seq reads relative to RNA-seq reads. As observed in our metaplot analysis, ribosome occupancy was significantly reduced within the 5′ UTR, at the TIS, and across the first coding exon in samples pulsed with DUX4 relative to untreated or IFNγ-treated cells, whereas fewer differential changes occurred across the CDS or 3′ UTR (Fig 3C and 3D and S2 Data). These changes are consistent with a decrease in translation initiation affecting the 5-prime mRNA region with relative preservation of ribosome footprints over the CDS, as suggested by the molecular mechanisms identified in Fig 2.

In cells treated with a DUX4 pulse+IFNγ versus IFNγ, 26.7% of transcripts showed significantly reduced TE at the 5′ UTR, 13.9% at the TIS, and 4.3% within the first exon (S7 Fig). These transcripts shared a large degree of overlap in enriched Gene Ontology (GO) terms, including processes involved in mRNA regulation, metabolism, translation, antigen processing, and MHC-I presentation (Fig 3E and S3 Data). Thus, Ribo-seq analysis suggests that a subset of RNAs and biological processes, such as antigen presentation pathways, are particularly sensitive to DUX4 translational suppression.

### Genome-wide polysome profiling identifies defects in translation initiation and elongation

Because ribosome footprinting reports the distribution of ribosome protected fragments rather than the ribosome abundance per transcript, it can be less effective at identifying differences in translation that parallel changes in protein expression. Therefore, we turned to classical polysome profiling to directly measure changes in ribosome density per mRNA using sucrose gradient-based isolation from MB135iDUX4 myoblasts treated with a pulse of DUX4+IFNγ harvested at 68 hours versus IFNγ alone. We pooled RNA fractions representing sub-polysome (40S-60S-80S), low polysome (1 to 3 ribosomes), and high polysome (>3 ribosomes) populations and performed RNA-seq analysis (S8A Fig). We initially measured changes in steady-state mRNA translation, excluding DUX4-altered gene expression. RNA abundance in each polysome fraction was determined relative to total input mRNA reads normalized to an internal spike-in (S8B Fig and S4 Data). The high-to-sub polysome (high/sub) ratio identified 5,800 genes that had decreased polysome association following a pulse of DUX4, consistent with a broad suppression of translation initiation, and 323 genes with increased polysome abundance (|log2FC>1|; p-adj<0.01) (Fig 3F and S4 Data).

GO analysis of the 323 genes enriched in the high polysome fraction showed an abundance of mRNAs characterized as ribosomal proteins and translation factors (Fig 3G). Many of these mRNAs contain 5-prime terminal oligopyrimidine (TOP) motifs [50] and are particularly sensitive to mTORC1 regulation of initiation factors [51,52] and eEF2K-eEF2 control of translation elongation [53]. Analysis of mRNAs with characterized TOP motifs [50] showed that TOP mRNAs remain associated with polysomes following a pulse of DUX4, while most other transcripts are depleted in the high polysome fraction (Fig 3H). This enrichment could reflect enhanced ribosome biogenesis used to poise cells for a rapid shift in translation rate upon recovery from DUX4 suppression. Conversely, mRNA enrichment in the high polysome fraction could result from stalled and accumulating ribosomes correlated with inhibited elongation and reduced protein expression. TOP mRNA-encoded ribosomal proteins RPL10A, RPL4, RPS6, and RPS15A were suppressed by DUX4 even though the mRNAs remain bound by polysomes (Fig 3I and 3J, and S8C Fig), consistent with inhibited translation elongation.

To elucidate DUX4 posttranscriptional suppression of MHC-I and iProteasome subunits specifically, we similarly assessed their mRNA high-to-sub polysome ratios. *HLA-A*, *HLA-B*, and *HLA-C* mRNAs showed reduced polysome association indicative of impaired translation initiation; however, *PSMB8*, *PSMB9*, and *PSMB10* mRNAs remained associated with polysomes, like TOP mRNAs (Fig 3I). Therefore, we propose that translation of MHC-I mRNAs is particularly sensitive to initiation defects, while a subset of mRNAs, including a subset of TOP mRNAs and iProteasome mRNAs, appear suppressed by DUX4 possibly through combinatorial inhibition of translation initiation and stalled elongation.

Previous analysis of the DUX4-induced transcriptome in muscle cells showed that DUX4 can both activate and inhibit genes to prevent myogenic differentiation [24,54,55]. This led us to question whether a pulse of DUX4 also had a prolonged suppressive effect on myogenesis. Interestingly, several early myogenic markers, including *ITGA7*, *PAX3*, *MEF2A*, *MEF2C*, and *MEF2D* mRNAs, showed a loss of polysome abundance and decreased protein levels indicative of reduced translation (Fig 3I and 3J). While the master regulator of skeletal myogenesis, *MYOD1*, showed no change in polysome abundance, and mRNAs encoding the muscle-specific gene *Desmin* had an increase in polysomes (Fig 3I), both were suppressed at the protein level following a pulse of DUX4 (Fig 3J). Thus, consistent with our molecular and Ribo-seq analysis, polysome profiling supports DUX4-induced translational suppression of broad classes of mRNAs, including those involved in antigen presentation and lineage determination.

### Translation of DUX4-induced mRNAs

**Fig 4:**
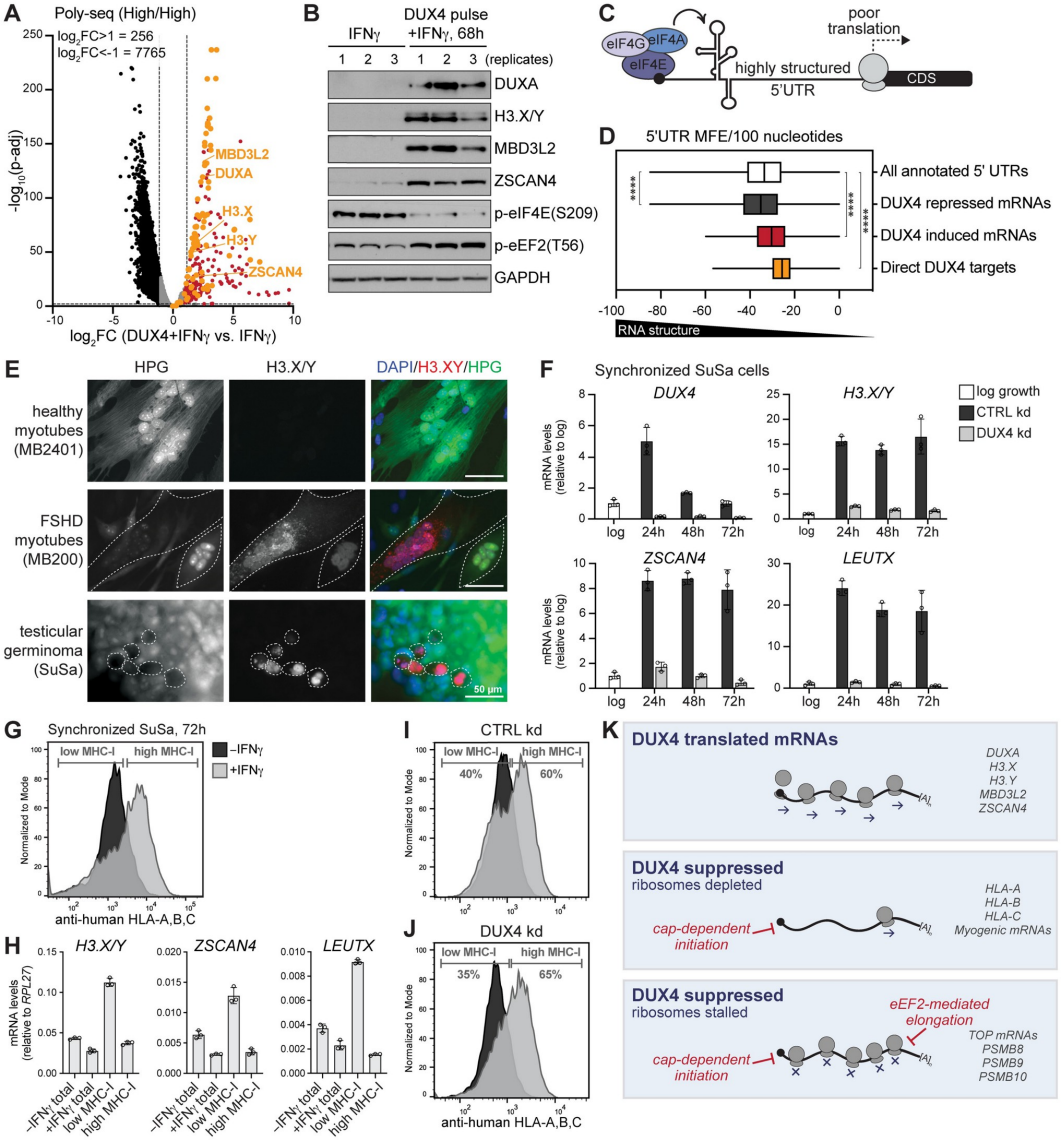
DUX4 orchestrates translational reprogramming through broad inhibition of translation concurrent with de novo translation of DUX4 target mRNAs. (**A**) Differential RNA-seq analysis of high polysome fractions (high/high). Volcano plot showing log2 fold-change of DUX4 pulse+IFNγ harvested at 68 hours versus IFNγ (significance defined as basemean>50, |log2FC>1|, p-adj<0.01); see S4 Data. Polysome-bound mRNAs up-regulated by DUX4 highlighted in red (*n =* 256 genes). Direct DUX4 target genes highlighted in orange (*n* = 70 genes). (**B**) Immunoblot analysis of total protein lysate harvested for polysome profiling samples representing biological replicates, *n* = 3. (**C**) Schematic of mRNA with structured 5′ UTR that impedes translation. (**D**) Box plot showing 5′ UTR analysis of predicted MFE per 100 nt, including all annotated mRNAs, a subset of direct DUX4 targets, mRNAs up- or down-regulated following a pulse of DUX4 (defined as mRNAs differentially expressed in (**A**)). See S5 Data for 5′ UTR sequences and analysis. Statistical comparisons were conducted using Mann–Whitney *U* test, **** *p* < 1 × 10^−8^. (**E**) Immunofluorescence of HPG Click-iT labeled nascent proteins in differentiated FSHD myotubes and SuSa cells costained for DUX4-target genes H3.X/Y. MB2401 myotubes serve as a control cell line that does not express DUX4 (scale bars, 50 μm). (**F**) RT-qPCR analysis of unsynchronized SuSa cells in log growth phase (log) relative to a time course following release from synchronization with gapmer-mediated CTRL or DUX4 kd. Data represent mean ± SD of biological replicates, *n* = 3; see S1 Data. (**G**) FACS analysis of MHC-I surface levels on SuSa cells 72 hours after synchronization and treated with and without IFNγ. (**H**) RT-qPCR analysis of SuSa cells treated with or without IFNγ and sorted based on high versus low MHC-I surface levels highlighted in (**G**); see S1 Data. (**I**, **J**) Flow cytometry analysis of MHC-I surface levels of SuSa cells 72 hours after synchronization and gapmer-mediated CTRL or DUX4 kd with and without IFNγ treatment. (**K**) Model of ribosome abundance and translation efficiency resulting from DUX4-induced inhibition of translation initiation and elongation. CTRL, control; DUX4, double homeobox protein 4; FACS, fluorescence-activated cell sorting; FSHD, facioscapulohumeral muscular dystrophy; HPG, L-homopropargylglycine; IFNγ, interferon gamma; kd, knockdown; MFE, minimum free energy; MHC-I, major histocompatibility complex class I; nt, nucleotide; RNA-seq, RNA sequencing; RT-qPCR, quantitative reverse transcription PCR; UTR, untranslated region.

Our previous studies showed that mRNAs transcriptionally induced by DUX4 are translated into protein [28]. Comparing the high polysome fractions of DUX4 pulse+IFNγ samples to IFNγ-treated samples provided a measure of how DUX4 changes the overall translatome and showed that DUX4-induced mRNAs were indeed associated with polysomes (Fig 4A and S4 Data). Thousands of mRNAs were reduced in the DUX4 pulse+IFNγ high polysome fraction (7,765 genes; log2FC<−1, p-adj<0.01); whereas 256 polysome-bound mRNAs were significantly up-regulated (log2FC>1, p-adj<0.01), many of which are well-characterized DUX4-target genes [56]. Immunoblot analysis confirmed that several DUX4-induced mRNAs in the high polysome fraction correlated with translation of these mRNAs (Fig 4B).

The enrichment of DUX4-induced mRNAs in the high polysome fraction might reflect their increased abundance following DUX4 expression or a relative resistance to the DUX4-mediated translational inhibition, or both. Thermodynamic stability and RNA secondary structures within the 5′ UTR of an mRNA can influence translation initiation rates of distinct transcripts in *cis* (Fig 4C), with higher predicted minimum free energy (MFE) showing increased translation efficiency [57,58]. The annotated 5′ UTRs of the 256 genes induced by DUX4 had a significantly higher predicted MFE per 100 nt relative to the average of all annotated 5′ UTRs, whereas the average MFE of 5′ UTRs belonging to the 7,765 genes repressed by DUX4 was significantly lower (Fig 4D). Furthermore, selective usage of alternative transcription start sites (TSS) have been shown to alter 5′ UTR sequences to influence cell type–specific protein synthesis [59]. We have previously observed that some DUX4-bound repetitive elements are co-opted to form alternative promoters for DUX4 target genes [22,56]. To account for noncanonical TSS and splicing events, we annotated the functional 5′ UTRs of 70 direct DUX4 targets based on RNA-seq alignment and published DUX4 ChIP peaks (S5 Data and S8D Fig). Indeed, DUX4 targets were predicted to have less structured 5′ UTRs on average (Fig 4D). Therefore, DUX4-induced mRNAs are predicted to be less susceptible to inhibition of translation initiation, which correlates with their observed increase in protein expression and polysome association.

### Expression of endogenous DUX4 correlates with translational suppression and reduced MHC-I surface antigens in cancer cells

To determine whether endogenous DUX4 expression alters mRNA translation similar to our DOX-inducible DUX4 model system, we used FSHD muscle cells and SuSa germinoma cells, 2 cell types previously shown to express DUX4 [3,21]. In both FSHD and SuSa cells, a rare population of cells stochastically express endogenous DUX4 at any given time. These cell types, like ESCs, exhibit cell-to-cell heterogeneity in culture, with only about 0.1% to 5% of cells expressing the DUX4 transcriptional program [3,16,23]. Metabolic labeling of de novo protein synthesis with HPG in FSHD myotubes and SuSa cells showed a dramatic reduction in HPG Click-iT signal in cells expressing DUX4-target genes H3.X and H3.Y (Fig 4E), demonstrating translation of DUX4-induced mRNAs in cells with otherwise broadly suppressed protein synthesis.

To study the downstream long-lived consequences of endogenous DUX4 expression in SuSa cells, we synchronized cells using a release from confluence culture protocol (see Methods). This resulted in a burst of endogenous DUX4 expression and prolonged expression of DUX4 target genes *H3*.*X/Y*, *ZSCAN4*, and *LEUTX*, and we showed that gapmer-mediated knockdown of *DUX4* abrogated target gene expression (Fig 4F). Fluorescence-activated cell sorting (FACS) of IFNγ-stimulated SuSa cells revealed that cells with low levels of MHC-I had elevated levels of DUX4 target gene expression relative to MHC-I high cells (Fig 4G- and 4H). Knockdown of endogenous DUX4 in SuSa cells rescued 5% of the MHC-I low population relative to control (Fig 4I and 4J), corresponding with the percentage of cells that expressed a brief pulse of DUX4 as evidenced by prolonged expression of DUX4 targets H3.X/Y (Fig 4E and 4F). Together, these results show that endogenous DUX4 expression in 2 independent cell types, FSHD muscle cells and SuSa germinoma cells, suppress protein synthesis and that this correlates with suppression of IFNγ-induced MHC-I expression in cancer cells. Our data demonstrate that DUX4 orchestrates broad inhibition of mRNA translation concurrent with de novo translation of DUX4-induced mRNAs (Fig 4K), effectively reprogramming the cellular translatome.

## Discussion

In this study, we have shown that brief expression of the developmental transcriptional factor DUX4 results in prolonged translational reprogramming of the cell. DUX4 expression drives an early embryonic gene program that facilitates a totipotent-like state [1,2,16]. Reprogramming cells to totipotency requires both the activation of a new gene expression program and suppression of existing gene products to erase the previous cellular identity. Broad suppression of protein synthesis is often accompanied by selective translation of mRNA networks during instances of cell stress and cellular reprogramming [60–63]. Critically, we found that DUX4 induced relatively broad translational regulation, where translation of many classes of mRNAs—including factors involved in antigen presentation, translation, and somatic cell lineage specification—were suppressed while DUX4-induced transcripts were translated. Thus, DUX4-induced transcription and downstream translational regulatory mechanisms ultimately result in reprogramming of protein synthesis, underscoring the importance of understanding DUX4 biology beyond its role as a transcriptional activator.

Many signaling pathways converge on the same key translation factors to rapidly control protein expression at both the initiation and the elongation stages. Building upon our previous observation of eIF2-alpha phosphorylation and changes to the proteome in DUX4-expressing myoblasts [28,35], we report novel effects of DUX4 involving the key regulators of translation initiation 4EBP1, eIF4E, and elongation factor eEF2. These factors are often modulated by intracellular and environmental cues, including nutrient and energy deprivation, cellular stress, hypoxia, and DNA damage. The exact intermediate mechanisms disrupting these translation factors downstream of DUX4 remain unknown. However, we show that multiple pathways are involved that may act redundantly. Complimentary to what we report here, recent studies have looked at early time points following continuous DUX4 expression in muscle cells and identified posttranscriptional regulatory mechanisms impacting protein phosphorylation, protein stability, nonsense mediated decay, and mRNA splicing [27,28,64,65].

Mechanisms underlying broad repression of translation and selective translation are often tightly coupled, and we argue that translational reprogramming is innate to the role of DUX4 as a developmental regulator. Recent work in mammalian preimplantation embryos optimized high-throughput ribosome profiling techniques for low-input applications [66–68]. These studies demonstrated a marked shift in the translatome associated with zygotic genome activation. As protein synthesis is an energetically expensive cellular process, we propose a model by which DUX4 initiates broad suppression of translation to reduce the cellular burden of protein synthesis while DUX4-induced mRNAs undergo translation to produce proteins vital for development. In line with this, we found that DUX4-induced mRNAs are predicted to have less structured 5′ UTRs, are associated with polysomes, and are up-regulated at the protein level. We propose that translational reprogramming by DUX4 facilitates translation of mRNAs promoting an early embryonic program when general protein synthesis is compromised.

We also provide evidence of decreased de novo protein synthesis in FSHD and cancer cells endogenously expressing DUX4. Similar expression profiles are found in a rare cell population of mouse ESCs that exhibit a 2C-like signature driven by the expression of mouse Dux, a functional ortholog of human DUX4 [12,69]. These 2CLCs have been associated with increased potency [12,70] and a global reduction of translation [13], including suppression of pluripotency proteins [14] and ribosomal proteins [71]. Like the transient attenuation of protein synthesis following a pulse of DUX4 in human cells, translational suppression in this rare population of mouse ESCs is transitory and protein synthesis resumes upon exit from the 2C-like state [14,71], highlighting the dynamic nature of molecular events as cells transition between states.

Like stem cells, tumors exhibit heterogeneity with certain cells undergoing dedifferentiation and reactivating developmental genes, giving rise to cells with expanded potential [72]. Protein synthesis is frequently dysregulated in cancer [73–77], and translational reprogramming is increasingly recognized as a contributor to tumor heterogeneity and adaptive plasticity [8,78]. Indeed, examples of translational control involving the ternary complex member eIF2-alpha [79,80] and subunits of the translation initiation complex eIF4F, particularly eIF4E [81], have been shown to regulate tumor immune escape. Additionally, the eukaryotic elongation factor 2 kinase (eEF2K), responsible for inhibitory eEF2 phosphorylation that impedes protein synthesis, is overexpressed in several cancers and associated with poor survival outcomes [82]. Our study extends these observations, as we find DUX4 disrupts many of these same translational regulators to modulate immune signaling.

In summary, our study demonstrates that transient expression of DUX4 reprograms the translatome through combined transcriptional and posttranscriptional control. Monumental shifts in the transcriptome and translatome occur during the initial stages of development as the fertilized egg transitions to the totipotent cells of the early embryo [5,83]. In human preimplantation embryos, ZGA is required for adequate suppression of the previous maternal program [6]. We provide molecular insight into how DUX4, a driver of ZGA, facilitates cellular reprogramming in somatic cells by controlling mRNA translation and suggest that it likely has a similar role during embryogenesis. We speculate that DUX4-expressing cancer cells might hijack these mechanisms to enhance cellular plasticity and create an immunosuppressive milieu, thereby promoting tumorigenesis and therapeutic resistance. This work provides new insights into cellular reprogramming and highlights opportunities for FSHD and cancer treatments targeting DUX4 and its downstream effectors. As a result, combining our knowledge of DUX4 biology in cancer, FSHD, and embryonic development will be critical to understand conserved pathways and to develop innovative therapeutic approaches.

## Materials and methods

### Cell culture

MB135 myoblasts were grown in Ham’s F-10 supplemented with 10% FBS, 1% penicillin/streptomycin, 10 ng/mL rhFGF, 1 μM dexamethasone, and 3 μg/mL puromycin as appropriate to maintain lines carrying the DUX4 transgene. SuSa cells were grown in RPMI 1640 supplemented with 10% FBS and 1% penicillin/streptomycin. Differentiation of FSHD myoblasts into myotubes was achieved by switching myoblast grown to confluence into DMEM, 1% penicillin/streptomycin, 10 μg/ml insulin, and 10 μg/ml transferrin for 48 hours. Pulsed MB135iDUX4 myoblasts were treated with 1 to 2 μg/mL DOX for 4 hours, rinsed with PBS, and fresh growth media added. MB135iDUX4 myoblasts with continuous DUX4 induction were treated with 1 μg/mL DOX for 20 hours. All cell types were stimulated with 50 ng/mL IFNγ where specified. MB135iDUX4 myoblasts were pulsed with DOX for 4 hours, incubated for 48 hours, supplemented with MG132 (0.5 μM), Bafilomycin A1 (0.5 μM), or ONX-0914 (200 nM) with the addition of 50 ng/mL IFNγ for 16 hours. MB135 myoblasts treated with mTOR inhibitors Everolimus or Torin2 (Sigma) were incubated in media supplemented with 10 to 100 nM inhibitor for 24 hours, followed by an additional 16-hour incubation in media resupplemented with inhibitor plus 50 ng/mL IFNγ. MB135 myoblasts were treated with 25 to 50 μM 4EGI-1 (Sigma) for 48 hours, followed by an additional 16-hour incubation in media resupplemented with 4EGI-1 plus 50 ng/mL IFNγ. MB135 myoblasts treated with cell stress–inducing reagents etoposide (1 to 5 μM), hydrogen peroxide (200 to 400 μM), or cobalt chloride (250 to 500 μM) were incubated for 24 hours, followed by an additional 16-hour incubation in media resupplemented with cell stress–inducing reagent plus 50 ng/mL IFNγ. All cell lines were cultured at 37°C in a humidified incubator supplied with 5% CO_2_. Cell culture reagents listed in S1 Table.

### CRISPR-Cas9 knockout generation

Generation of MB135iDUX4 PKR KO myoblasts was achieved using a guide RNA (gRNA) sequence targeting *EIF2AK2* [84] cloned into the Cas9(BB)-2A-GFP plasmid (Addgene). MB135iDUX4 myoblasts were transfected with this construct using Lipofectamine 3000 Reagent (Invitrogen) according to the manufacturer protocol and incubated for 1.5 days. Clonal cells lines were isolated using fluorescence activated cell sorting. Individual PKR KO clones were screened using immunoblot analysis, and mutant alleles were validated with Sanger sequencing. Cloning and sequencing primers listed in S1 Table.

### Starvation-induced cell cycle synchronization

SuSa cells were seeded at 90% confluence on 0.1% gelatin-coated plates and incubated for 1 week at 37°C and 5% CO_2_. Cells were supplemented with fresh growth media and incubated for 3 to 4 hours to release from synchronization, lifted using trypsin, seeded onto gelatin-coated plates at 30% confluence, treated with or without IFNγ for the last 16 hours, and harvested at terminal time points of 24, 48, or 72 hours for downstream quantitative reverse transcription PCR (RT-qPCR), IF, or flow cytometry analysis. For flow cytometry, cells were stained with BV605 anti-human HLA-A,B,C Antibody (BioLegend #311432), sorted using BD FACS Aria II, or analyzed using BD LSRFortessa X-50, paired with BD FACSDiva software. Data were analyzed using FlowJo V10.5.3.

### siRNA and gapmer transfections

Transfections of siRNAs into myoblasts were carried out using Lipofectamine RNAiMAX (Invitrogen) according to the manufacturer’s protocol. A double transfection protocol was followed with MB135iDUX4ca pulse experimental conditions to ensure prolonged depletion of target proteins, where cells were transfected 20 hours before and 20 hours after a 4-hour pulse of DOX. SuSa cells were synchronized and released as described above and reverse transfected with 1 μl/mL Lipofectamine RNAiMAX and 25 pmol/mL of either a pool of 2 DUX4-targeting gapmers [85] or a nontargeting control gapmer. Cells were incubated with gapmers for 24 hours. siRNA and gapmer sequences are listed in S1 Table.

### Immunoblotting

Protein samples were harvested in RIPA buffer [150 mM NaCl, 1% NP-40, 0.5% Na-deoxycholate, 1% SDS, 25 mM Tris–HCl (pH7.4)] supplemented with protease and phosphatase inhibitor tablets, followed by sonication in Diagenode Bioruptor. Lysate was cleared by centrifugation at 16,000×*g* and quantified using a Pierce BCA assay. Samples were run on NuPAGE precast polyacrylamide gels and transferred to PVDF membrane. Membranes were blocked in PBS containing 0.1% Tween-20 and 5% nonfat dry milk before overnight incubation at 4°C with primary antibodies. Membranes were incubated with horseradish peroxidase–conjugated secondary antibodies for 1 to 2 hours at room temperature, and SuperSignal chemiluminescent substrate was used for detection on film. Membranes were stripped with Restore Western Blot Stripping Buffer. Antibodies and reagents listed in S1 Table.

### Subcellular fractionation

MB135iDUX4 myoblasts were pulsed with DOX for 4 hours, incubated for 24 hours, supplemented with 50 ng/mL IFNγ for 16 hours, and harvested at a terminal time point of 44 hours. Samples divided for whole-cell lysate (WCL) and subcellular fractionation were suspended in 300 μL ice-cold Cyto-lysis buffer (10 mM Tris (pH 7.4), 10 mM NaCl, 0.2% NP-40, 1 mM DTT in nuclease-free water). Subcellular fractionation samples were centrifuged at 650 RCF to pellet nuclei, while cytoplasmic lysate remained in the supernatant. WCL, cytoplasmic, and nuclear RNA and protein were harvested for RT-qPCR and immunoblotting, respectively.

### RT-qPCR

Total RNA was isolated using the NucleoSpin RNA kit according to manufacturer instructions. Isolated RNA was treated with Amp-grade DNase I, heat inactivated in the presence of EDTA, and reverse transcribed into cDNA using SuperScript IV First-Strand Synthesis System following the manufacturer’s protocol. Quantitative PCR was carried out on a QuantStudio 7 Flex using iTaq SYBR Green Supermix. Primers listed in S1 Table.

### m7GTP cap-binding assay

MB135iDUX4 myoblasts were treated with and without a 4-hour pulse of DOX and harvested at a terminal time point of 68 hours. Cells were lysed in cap binding buffer [10 mM Tris–HCl (pH 7.5), 140 mM KCl, 4 mM MgCl2, 1 mM DTT, 1 mM EDTA, 1% NP-40] supplemented with protease and phosphatase inhibitor cocktails, incubated on ice for 30 minutes, and lysate cleared at 12,000 rpm for 30 minutes at 4°C. Soluble lysate was quantified with Pierce BCA assay and diluted in cap binding buffer without NP-40 to bring final concentration to 0.5 mg/mL in 0.5% NP-40. Around 50 μl of prewashed 7-methyl-GTP-Sepharose bead slurry was added to 400 μg protein and incubated at 4°C for 1 hour. Samples were centrifuged at 5,000 rpm for 5 minutes at 4°C, washed twice with cap binding buffer containing 0.5% NP-40 and twice with PBS. Beads were suspended in NuPAGE LDS Buffer and incubated at 95°C for 10 minutes to elute associated proteins. Reagents listed in S1 Table.

### [35S] Radiolabeling

Cells were treated with and without a 4-hour pulse of DOX. Eight hours prior to harvest, cells were incubated in DMEM depleted for methionine and cysteine supplemented with 90 microcurie 35S-methionine/cysteine and 50 ng/mL IFNγ. Protein samples were harvested and run on NuPAGE gels as previously described. Gels were stained with InstantBlue Coomassie, dried on whatman paper, exposed to phosphor screen, imaged on Typhoon Trio imager, and analyzed with ImageQuant. Reagents listed in S1 Table.

### HPG Click-iT and immunofluorescence

Cells were incubated for 30 minutes in DMEM or RPMI media depleted for methionine and cysteine, followed by a 1-hour incubation in methionine-depleted media supplemented with 200 μM HPG. Cells were fixed with 4% paraformaldehyde for 10 minutes, permeabilized 0.5% TritonX-100, and stained with Click-iT HPG Alexa Fluor 488 Protein Synthesis Assay Kit according to manufacturer’s protocol. Reagents listed in S1 Table. Samples were incubated with primary antibodies at 4°C overnight, followed by incubation with fluorescently conjugated secondary antibodies for 1 hour at room temperature, and counterstained with DAPI. Plates were imaged using an immersion lens, Zeiss Axiophot fluorescent microscope, AxioCam MRc digital camera, and AxioVision 4.6 software. For flow cytometry, cells were analyzed using BD LSRFortessa X-50 with BD FACSDiva software. Data were analyzed using FlowJo v10.5.3. Antibodies and reagents listed in S1 Table.

### Ribosome footprinting

Ribo-seq was performed as described previously by Calviello and colleagues [86] using two 70% confluent 15 cm plates of MB135iDUX4 myoblasts per treatment condition (*n =* 3). Ribosome complexes were isolated using MicroSpin S-400 HR Columns and RNA extracted using the Direct-zol RNA Miniprep Kit. The rRNA Removal Mix–Gold component of Illumina’s TruSeq Stranded Total RNA Library Prep Gold kit was used to deplete rRNAs. RPFs were isolated by running samples on a 15% TBE-Urea gel with 10 bp DNA Ladder and Marker-27nt and Marker-30nt (see S1 Table for sequences). Gels were stained with SYBR Gold and RNA fragments 27 to 30 nt were isolated. RNA samples were diluted to equal input concentrations; libraries were prepared using the NEXTflex Small RNA-Seq Kit v3 following the manufacturer’s instructions and sequenced using 50 bp paired-end sequencing on the Illumina NextSeq platform by the Fred Hutchinson Cancer Center Genomics Core. Reagents listed in S1 Table.

### Polysome fractionation

Polysome profiling was performed using three 70% confluent 15 cm plates of MB135iDUX4 myoblasts per treatment condition (*n* = 3). MB135iDUX4 myoblasts were pulsed with or without DOX for 4 hours, incubated for 48 hours, supplemented with 50 ng/mL IFNγ for 16 hours, and harvested at a terminal time point of 68 hours. To harvest, culture medium was supplemented with 100 μg/mL CHX and cells were incubated at 37°C for 10 minutes. Media was aspirated and each 15 cm plate of adherent cells was rinsed with 25 mL ice-cold PBS supplemented with 100 μg/mL CHX, lifted with 0.25% Trypsin–EDTA supplemented with CHX, and resuspended in growth media supplemented with CHX. Cells were pelleted and flash frozen in liquid nitrogen. Cells were lysed with Polysome Lysis Buffer (10 mM Tris (pH 8), 140 mM NaCl, 7.5 mM MgCl2, 0.25% NP-40, 0.1% Triton X-100, 150 μg/mL CHX, 20 mM DTT, 640 U/mL SUPERase-In RNase Inhibitor) and clarified before quantification with the Bio-Rad Protein Assay. About 1.5 mg of clarified lysate was loaded onto a 10% to 50% sucrose gradient prepared in DEPC-treated water with 25 mM Tris (pH 7.4), 25 mM NaCl, 5 mM MgCl2, 100 μg/mL heparin, and 2 mM DTT. Gradients were fractionated using a Biocomp Piston Gradient Fractionator. Samples were resuspended in Trizol and Drosophila S2 cells were added as an internal spike-in control. RNA was extracted using the Direct-zol RNA Miniprep Kit. Relative mRNA abundance for each sample was normalized to Drosophila spike-in to account for differences in RNA extraction efficiency. RNA-seq libraries were prepared using the Illumina TruSeq RNA Sample Prep v2 Kit and sequenced using 50 bp paired-end sequencing on the Illumina NextSeq platform by the Fred Hutchinson Cancer Center Genomics Core. Reagents listed in S1 Table.

### RNA-seq data analysis

To preprocess the RNA-seq data, we used Trimmomatic to trim the 3′ adapter sequence and aligned the trimmed reads to GRCh38 (p13) using R*subread*. We counted mapped reads that overlapped with exons of gene features using the *summarizeOverlaps* function of Bioconductor’s *GenomicAlignments* package [87] with the IntersectionStrict mode. The gene features were annotated by Gencode v35. We applied DESeq2 for gene expression normalization, log transformation, and differential analysis on different comparisons between treatment conditions.

### Ribo-seq data analysis

We performed preprocessing and quality assessment for Ribo-seq data in the following manner: (1) Clipped the 3′ adapter sequence and trim the first and last 4 bases from the adapter-clipped reads (*cutadapt*). (2) Removed rRNA and other small RNA such as tRNA and snoRNA. We customized the rRNA reference genome from the RNA central database (https://rnacentral.org/) and then used *Bowtie2* to align the trimmed reads against it; the unmapped reads are the desirable RPFs. (3) Aligned RPFs to the GRCh38 (p.13) genome built by *STAR*. (4) Assessed the quality and size of RPFs using Bioconductor’s *ribosomeProfilingQC* package. (5) Profiled ribosome footprints using *ribosomeProfilingQC* and the Gencode (v35) annotation. We computed the p-sites counts of RPFs of the dominant length (26 to 29 nt) for 5 different genomic features including the 5′ UTRs, translation start sites (TIS; 13 nt extended up/downstream from the start codon), CDS, and 3′ UTRs (S6 Fig). We formulated the treatment effects on translation efficiency as changes in Ribo-seq reads between 2 different treatments relative to RNA-seq reads. The null hypothesis is defined as

$${Η}_{0}:\left\{{\text{log}}_{2}\frac{treated}{untreated}{|}_{Ribo-seq}-{\text{log}}_{2}\frac{treated}{untreated}{|}_{RNA-seq}\right\}=0.$$

We performed hypothesis tests on 5 different genomic features using DESeq2. S7 Fig depicts the flow of the differential analysis and specifies the dynamic nonspecific filtering prior to *DESeq2* for different genomic features. To only analyze steady-state genes, DUX4-altered genes (defined by |log2(DUX pulse/untreated) >1| and adjusted *p*-value<0.05 in RNA-seq samples) and IFNγ-altered genes (defined by |log2(IFNγ/untreated) >1| and adjusted *p*-value<0.05 in RNA-seq samples) were excluded from differential analysis. We used Bioconductor’s *goseq* package to perform GO analysis on the transcripts showing down-regulated translational changes (FDR = 0.05).

### Polysome-seq data analysis

Polysome RNA-Seq reads were quality checked with *FastQC*, trimmed using *Trim Galore*!, and aligned to the human reference genome GRCh38 p13 as well as the DM6 Drosophila reference genome using *Rsubread*. Reads were mapped to the Gencode v35 reference annotation using *FeatureCounts*. The human counts were normalized using both the DM6 and GRCh38 library sizes, log transformed, and analyzed for differential gene expression using *DESeq2*. Differential mRNA polysome association was analyzed in 3 different ways:

(1) Polysome gradient relative to total mRNA: We formulated the changes of mRNA abundance in sub-polysome, low polysome, and high polysome fractions between 2 different treatments as a ratio of ratios, calculating the abundance of mRNA in each fraction relative to total RNA-seq. DUX4-altered genes (defined as genes with |log2FC>2| and adjusted *p*-value<0.01 in DUX4 pulse+IFNγ versus IFNγ sample inputs) were excluded from the differential analysis. The null hypothesis is defined as
$${Η}_{0}:\left\{{\text{log}}_{2}\frac{fraction}{total\;mRNA}{|}_{DUX4+IFNg}-{\text{log}}_{2}\frac{fraction}{total\;mRNA}{|}_{IFNg}\right\}=0.$$
(2) High-to-sub polysome ratio: We formulated the changes of mRNA abundance in the high polysome fractions relative to the sub-polysome fraction between 2 different treatments as a ratio of ratios. DUX4-altered genes were excluded from the differential analysis. The null hypothesis is defined as
$${Η}_{0}:\left\{{\text{log}}_{2}\frac{high\;polysome}{sub-polysome}{|}_{DUX4+IFNg}-{\text{log}}_{2}\frac{high\;polysome}{sub-polysome}{|}_{IFNg}\right\}=0.$$
(3) Differential gene expression in high polysome fraction: We formulated the changes of mRNA abundance in polysome fractions between 2 different treatment conditions. DUX4-altered genes include in analysis. The null hypothesis is defined as
$${Η}_{0}:\left\{{\text{log}}_{2}\frac{DUX4+IFNg\;high\;polysome}{IFNg\;high\;polysome}\right\}=0.$$

### 5′ UTR analysis

Annotated 5′ UTR coordinates were obtained from Gencode v35, and the underlying sequences were extracted from the GRCh38 patch 13 genome using AGAT. Genes were filtered to only include transcripts expressed in MB135iDUX4 myoblasts expressing DUX4 for downstream 5′ UTR analysis, and duplicate sequences and 5′ UTRs with less than 4 nt were excluded. Alternative 5′ UTRs of direct DUX4 target genes were annotated using RNA-seq alignments from the human myoblast inducible DUX4 model (GSE85461) (see S5 Data). *RNAfold* from ViennaRNA package [88] was used to predict MFE.

## Supporting information

S1 Fig Subcellular mRNA localization via cell fractionation. (**A**) Immunoblot analysis of DUX4 targets H3.X/Y/Z, MHC-I, iProteasome subunits PSMB9 and PSMB10, and localization controls GAPDH (cytoplasmic) and Histone H3 (nuclear) after nuclear and cytoplasmic fractionation. MB135iDUX4 myoblasts were treated with or without DOX for 4 hours, and 24 hours later stimulated with or without IFNγ 16 hours; harvested cells 44 hours post-DOX treatment (DOX pulse, 44 hours). (**B**) RT-qPCR analysis shows no difference in mRNA localization after a pulse of DOX. Proper localization of *RPL27* (cytoplasmic) and *HSATII* (nuclear) was observed. Data represent mean ± SD; see S1 Data. C, cytoplasmic fraction; DOX, doxycycline; DUX4, double homeobox protein 4; IFNγ, interferon gamma; MHC-I, major histocompatibility complex class I; N, nuclear fraction; RT-qPCR, quantitative reverse transcription PCR; WCL, whole-cell lysate.
(TIF)

S2 Fig Treatment with MG132 or Bafilomycin does not rescue DUX4-induced protein suppression of MHC-I and the iProteasome. (**A**) Immunoblot analysis of MB135iDUX4 myoblasts treated with or without a 4-hour pulse of DOX, incubated for 48 hours, stimulated with or without IFNγ, and treated with DMSO, 0.5 μM MG132, or 0.5 μM Baf for an additional 16 hours; harvested cells 68 hours post-DOX treatment (DOX pulse, 68 hours). DUXA is a DUX4-activated target gene. GAPDH serves as loading control. (**B**) RT-qPCR analysis shows no effect of MG132 or Baf on relative mRNA levels of housekeeping gene *RPL27*, DUX4-target genes *DUXA* and *H3*.*X/Y*, MHC-I subunits *HLA-A*, *HLA-B*, *HLA-C*, or iProteasome subunits *PSMB8*, *PSMB9*, and *PSMB10*. Data represent mean ± SD; see S1 Data. Baf, Bafilomycin; DOX, doxycycline; DUX4, double homeobox protein 4; IFNγ, interferon gamma; MHC-I, major histocompatibility complex class I; RT-qPCR, quantitative reverse transcription PCR.
(TIF)

S3 Fig MHC-I protein stability does not require iProteasome catalytic subunits PSMB8 or PSMB9. (**A**) Immunoblot analysis of MB135 myoblasts treated with siRNAs targeting PSMB8, PSMB9, or nontargeting siCTRL, with and without 16-hour IFNγ treatment. GAPDH serves as loading control. (**B**) Immunoblot analysis of MB135iDUX4 myoblasts treated with DMSO or PSMB8 inhibitor ONX-0914. Cells were treated with a 4-hour pulse of DOX, incubated for 48 hours, followed by treatment with 200 nM ONX-0914 and IFNγ for the terminal 16 hours; harvested cells 68 hours post-DOX treatment (DOX pulse, 68 hours). GAPDH serves as loading control. (**C**) Genotype of polyclonal CRISPR-Cas9 engineered PKR KO in MB135iDUX cells. (**D**) Immunoblot analysis of MB135iDUX4 myoblasts expressing WT PKR (left) or PKR KO (right) stimulated with IFNγ following continuous DUX4 induction or a pulse of DUX4 harvested at 68 hours. GAPDH serves as loading control. (**E**) Quantification of eIF2-alpha phosphorylation levels in (**D**) using densitometric analysis normalized to GAPDH and graphed as fold change relative untreated samples; see S1 Data. DOX, doxycycline; IFNγ, interferon gamma; KO, knockout; MHC-I, major histocompatibility complex class I; siCTRL, siRNA Control; WT, wild-type.
(TIF)

S4 Fig Validation of IFNγ-induced mRNA expression levels with Everolimus, Torin2, and 4EGI-1 treatments. (**A**) RT-qPCR analysis shows no statically significant effect of Everolimus or Torin2, or (**B**) 4EGI-1 treatment, on the relative mRNA levels of MHC-I subunits *HLA-A*, *HLA-B*, *HLA-C*, or iProteasome subunits *PSMB8*, *PSMB9*, and *PSMB10* induced by IFNγ treatment in MB135 myoblasts. Data were normalized to *RPL27* then graphed relative to IFNγ-treated samples and represent mean ± SD; see S1 Data. (**C**) Immunoblot analysis of MB135 myoblasts treated with the eIF4E/eIF4G inhibitor 4EGI-1, with and without IFNγ. Beta-actin serves as loading control. IFNγ, interferon gamma; MHC-I, major histocompatibility complex class I; RT-qPCR, quantitative reverse transcription PCR.
(TIF)

S5 Fig Oxidative stress, DNA damage, and hypoxia-induced cell stress pathways moderately suppress MHC-I and PSMB9. (**A**) Immunoblot analysis of MB135 myoblasts treated with hydrogen peroxide (H_2_O_2_) to induce oxidative stress, (**B**) etoposide to induce DNA damage, or (**C**) cobalt chloride (CoCl_2_) to mimic hypoxia. Cells were treated with stress-inducing reagents for 24 hours, followed by a 16-hour incubation in media resupplemented with cell stress–inducing reagent plus IFNγ. Beta-actin serves as loading control. IFNγ, interferon gamma; MHC-I, major histocompatibility complex class I.
(TIF)

S6 Fig Quality control analysis of Ribo-seq data. (**A**) PCA of gene expression in Ribo-seq biological replicates, *n =* 3; see S1 Data. (**B**) Length distribution of RPFs; see S1 Data. All downstream analysis was restricted to the dominant fragment size of 26–29 nt. (**C**) Metagene coverage of P-sites in 3 different reading frames (green, red, blue) over 5′ UTR, TIS, CDS, and 3′ UTR regions; data represent the average of biological replicates for each treatment condition, *n* = 3. CDS, coding sequence; nt, nucleotide; PCA, principal component analysis; RPF, ribosome-protected fragment; TIS, translation initiation site.
(TIF)

S7 Fig Genome-wide quantification of ribosome-protected mRNA fragments using RNA sequencing (RNA-seq) and ribosome profiling (Ribo-seq). (**A**) Workflow of differential analysis and filtering prior to DESeq2 analysis to determine translation efficiency at genomic features. (**B**) Scatter plots of reads aligning to annotated genomic features at the transcript or gene level as indicated. Log2 fold-change values represent the average of biological replicates for DUX4 pulse+IFNγ condition relative to IFNγ treatment alone in MB135iDUX4 myoblasts, *n* = 3. Translationally up-regulated mRNAs (red) and translationally down-regulated mRNAs (blue) are highlighted (|log2FC>1|, p-adj<0.05, *n* = 3). DUX4, double homeobox protein 4; IFNγ, interferon gamma; RNA-seq, RNA sequencing.
(TIF)

S8 Fig Polysome profiling and 5′ UTR analysis. (**A**) Absorbance at 254 nm across a density gradient fractionation system. Traces represent biological replicates for each treatment condition, *n* = 3 (black = IFNγ; gray = DUX4 pulse+IFNγ harvested at 68 hours). (**B**) Differential RNA-seq analysis of sub-, low, and high polysome fractions relative to total mRNA levels. Volcano plot showing log2 fold-change differential abundance in cells treated with DUX4 pulse+IFNγ versus IFNγ (significance defined as basemean>50, |log2FC>1|, p-adj<0.01, *n* = 3); see S4 Data. (**C**) 5′ UTR sequences of select TOP mRNAs (blue = TOP motif). (**D**) Two representative direct DUX4-target genes with alternative 5′ UTRs based on RNA-seq alignment shown relative to the annotated transcripts. DUX4, double homeobox protein 4; IFNγ, interferon gamma; RNA-seq, RNA sequencing; TOP, terminal oligopyrimidine.
(TIF)

S1 Data Source Data for Figs 1C, 2E, 3B, 4F, 4H, S1B, S2B, S3E, S4A, S4B, S6A and S6B. (XLSX)

S2 Data Differential analysis of Ribo-seq and RNA-seq read counts by genomic feature. Source Data for Fig 3D.
(XLSX)

S3 Data GO analysis of mRNAs with reduced translation efficiency in 5′ region. Source data for Fig 3E.
(XLSX)

S4 Data Differential RNA-seq analysis of polysome profiling. Source data for Figs 3F, 3H, 3I and 4A, and S8B.
(XLSB)

S5 Data Characterized 5′ UTRs and MFE. Source data for Fig 4D.
(XLSX)

S1 Raw Images Uncropped western blots and flow cytometry gates. (PDF)

S1 Table Oligonucleotide sequences, reagents, and resources used in this study. (XLSB)

## Abbreviations

2CLC: 2-cell-like cell
4EBP1: 4E-binding protein 1
DOX: doxycycline
dsRNA: double-stranded RNA
DUX4: double homeobox protein 4
eEF2: eukaryotic elongation factor 2
eEF2K: eukaryotic elongation factor 2 kinase
eIF4E: eukaryotic initiation factor 4E
ESC: embryonic stem cell
FACS: fluorescence-activated cell sorting
FSHD: facioscapulohumeral muscular dystrophy
GO: Gene Ontology
HPG: L-homopropargylglycine
IFNγ: interferon gamma
iPSC: induced pluripotent stem cell
ISG: interferon-stimulated gene
KO: knockout
m^7^GTP: 7-methylguaniosine 5′-triphosphate
MFE: minimum free energy
MHC-I: major histocompatibility complex class I
mTORC1: mechanistic target of rapamycin complex 1
nt: nucleotide
RNA-seq: RNA sequencing
RPF: ribosome-protected fragment
TE: translational efficiency
TIS: translation initiation site
TOP: terminal oligopyrimidine
TSS: transcription start site
WCL: whole-cell lysate
WT: wild-type
ZGA: zygotic genome activation
