# Supplementary figures and images for: The transcription factor DUX4 orchestrates translational reprogramming by broadly suppressing translation efficiency and promoting expression of DUX4-induced mRNAs
Source: PLoS Biol. 2023 Sep 25;21(9):e3002317. doi: 10.1371/journal.pbio.3002317 (PMC10553841; doi:10.1371/journal.pbio.3002317)

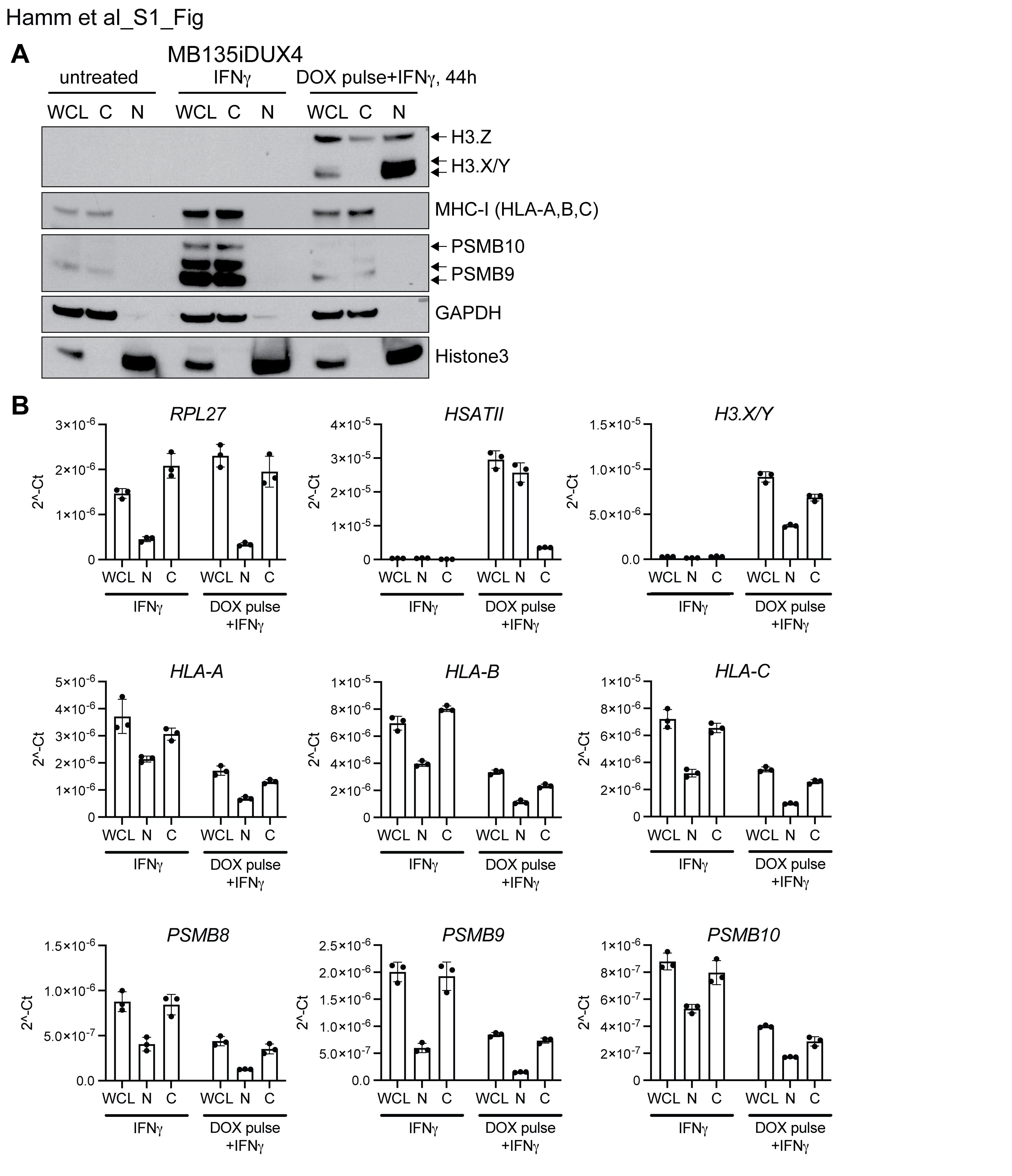

Supplement: S1 Fig — (A) Immunoblot analysis of DUX4 targets H3.X/Y/Z, MHC-I, iProteasome subunits PSMB9 and PSMB10, and localization controls GAPDH (cytoplasmic) and Histone H3 (nuclear) after nuclear and cytoplasmic fractionation. MB135iDUX4 myoblasts were treated with or without DOX for 4 hours, and 24 hours later stimulated with or without IFNγ 16 hours; harvested cells 44 hours post-DOX treatment (DOX pulse, 44 hours). (B) RT-qPCR analysis shows no difference in mRNA localization after a pulse of DOX. Proper localization of RPL27 (cytoplasmic) and HSATII (nuclear) was observed. Data represent mean ± SD; see S1 Data. C, cytoplasmic fraction; DOX, doxycycline; DUX4, double homeobox protein 4; IFNγ, interferon gamma; MHC-I, major histocompatibility complex class I; N, nuclear fraction; RT-qPCR, quantitative reverse transcription PCR; WCL, whole-cell lysate. (TIF) [file pbio.3002317.s001.tif]

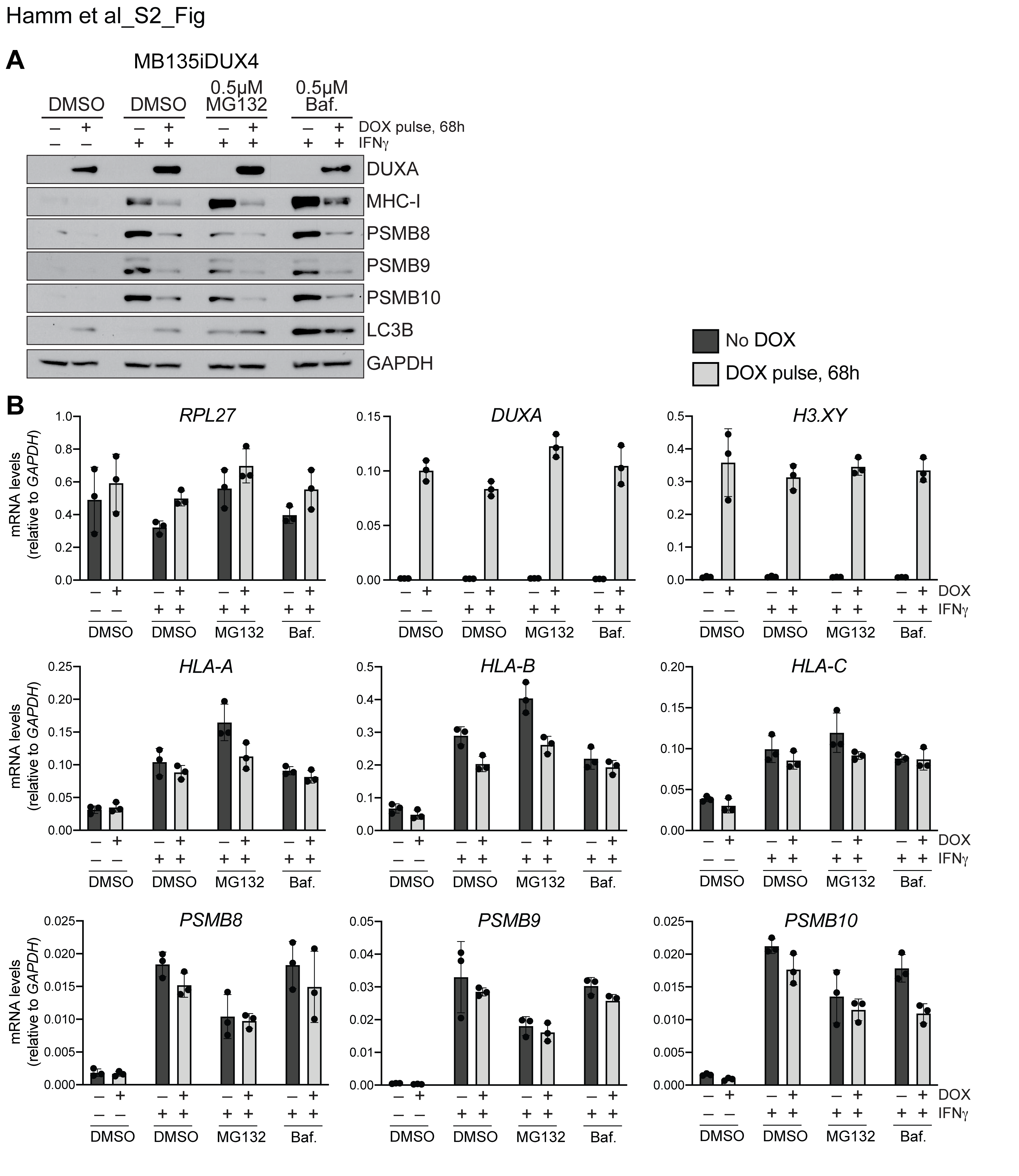

Supplement: S2 Fig — (A) Immunoblot analysis of MB135iDUX4 myoblasts treated with or without a 4-hour pulse of DOX, incubated for 48 hours, stimulated with or without IFNγ, and treated with DMSO, 0.5 μM MG132, or 0.5 μM Baf for an additional 16 hours; harvested cells 68 hours post-DOX treatment (DOX pulse, 68 hours). DUXA is a DUX4-activated target gene. GAPDH serves as loading control. (B) RT-qPCR analysis shows no effect of MG132 or Baf on relative mRNA levels of housekeeping gene RPL27, DUX4-target genes DUXA and H3.X/Y, MHC-I subunits HLA-A, HLA-B, HLA-C, or iProteasome subunits PSMB8, PSMB9, and PSMB10. Data represent mean ± SD; see S1 Data. Baf, Bafilomycin; DOX, doxycycline; DUX4, double homeobox protein 4; IFNγ, interferon gamma; MHC-I, major histocompatibility complex class I; RT-qPCR, quantitative reverse transcription PCR. (TIF) [file pbio.3002317.s002.tif]

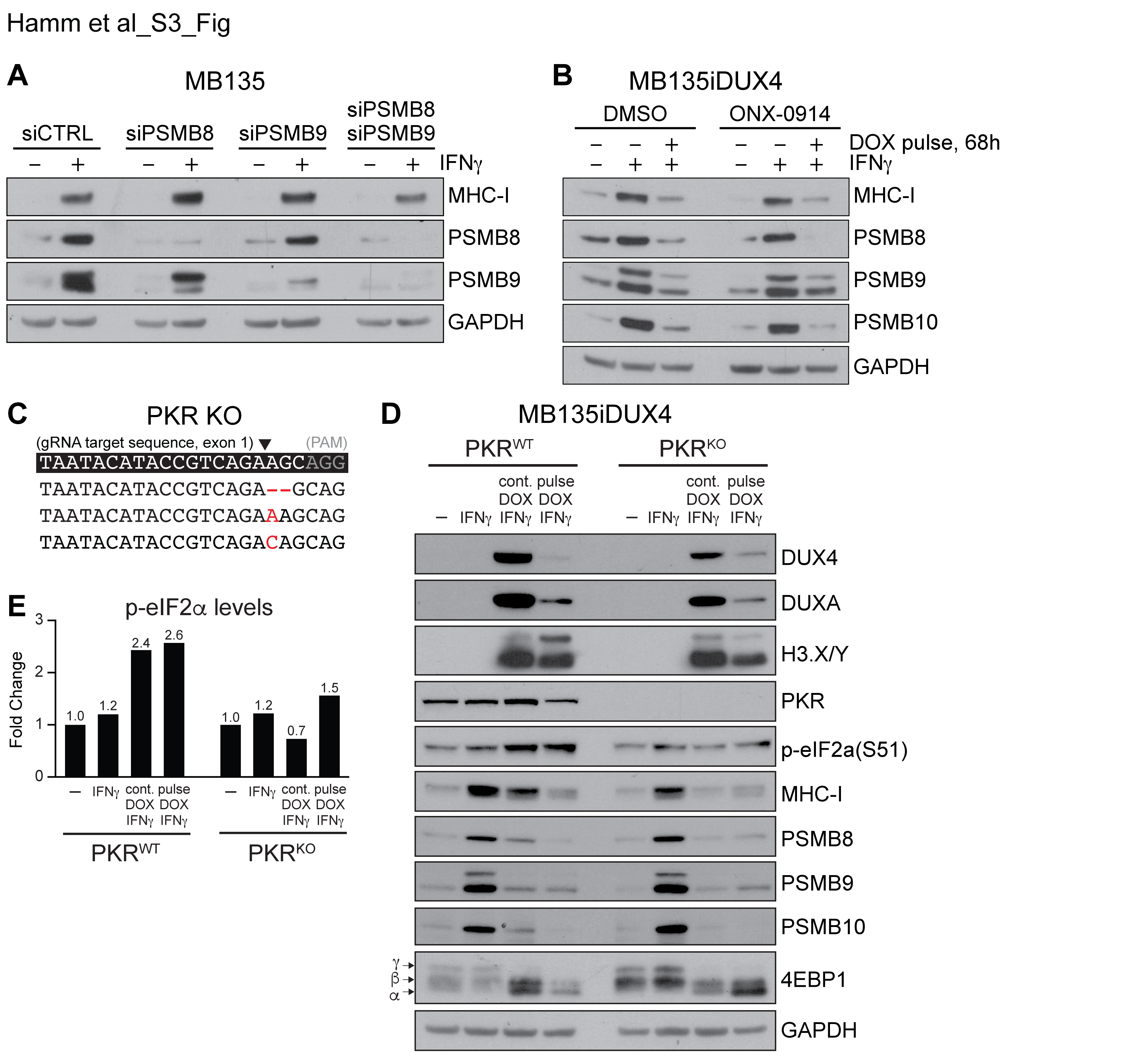

Supplement: S3 Fig — (A) Immunoblot analysis of MB135 myoblasts treated with siRNAs targeting PSMB8, PSMB9, or nontargeting siCTRL, with and without 16-hour IFNγ treatment. GAPDH serves as loading control. (B) Immunoblot analysis of MB135iDUX4 myoblasts treated with DMSO or PSMB8 inhibitor ONX-0914. Cells were treated with a 4-hour pulse of DOX, incubated for 48 hours, followed by treatment with 200 nM ONX-0914 and IFNγ for the terminal 16 hours; harvested cells 68 hours post-DOX treatment (DOX pulse, 68 hours). GAPDH serves as loading control. (C) Genotype of polyclonal CRISPR-Cas9 engineered PKR KO in MB135iDUX cells. (D) Immunoblot analysis of MB135iDUX4 myoblasts expressing WT PKR (left) or PKR KO (right) stimulated with IFNγ following continuous DUX4 induction or a pulse of DUX4 harvested at 68 hours. GAPDH serves as loading control. (E) Quantification of eIF2-alpha phosphorylation levels in (D) using densitometric analysis normalized to GAPDH and graphed as fold change relative untreated samples; see S1 Data. DOX, doxycycline; IFNγ, interferon gamma; KO, knockout; MHC-I, major histocompatibility complex class I; siCTRL, siRNA Control; WT, wild-type. (TIF) [file pbio.3002317.s003.tif]

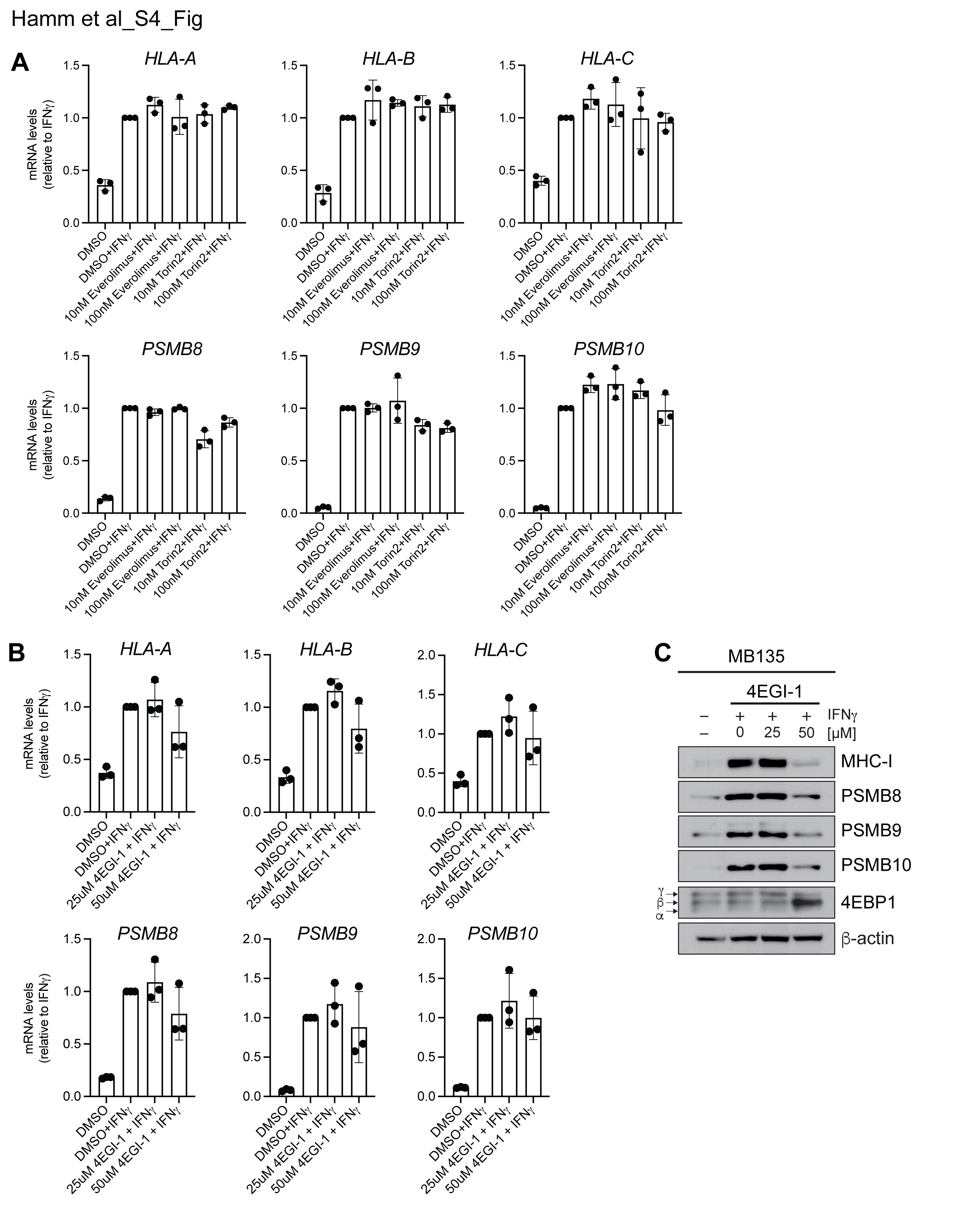

Supplement: S4 Fig — (A) RT-qPCR analysis shows no statically significant effect of Everolimus or Torin2, or (B) 4EGI-1 treatment, on the relative mRNA levels of MHC-I subunits HLA-A, HLA-B, HLA-C, or iProteasome subunits PSMB8, PSMB9, and PSMB10 induced by IFNγ treatment in MB135 myoblasts. Data were normalized to RPL27 then graphed relative to IFNγ-treated samples and represent mean ± SD; see S1 Data. (C) Immunoblot analysis of MB135 myoblasts treated with the eIF4E/eIF4G inhibitor 4EGI-1, with and without IFNγ. Beta-actin serves as loading control. IFNγ, interferon gamma; MHC-I, major histocompatibility complex class I; RT-qPCR, quantitative reverse transcription PCR. (TIF) [file pbio.3002317.s004.tif]

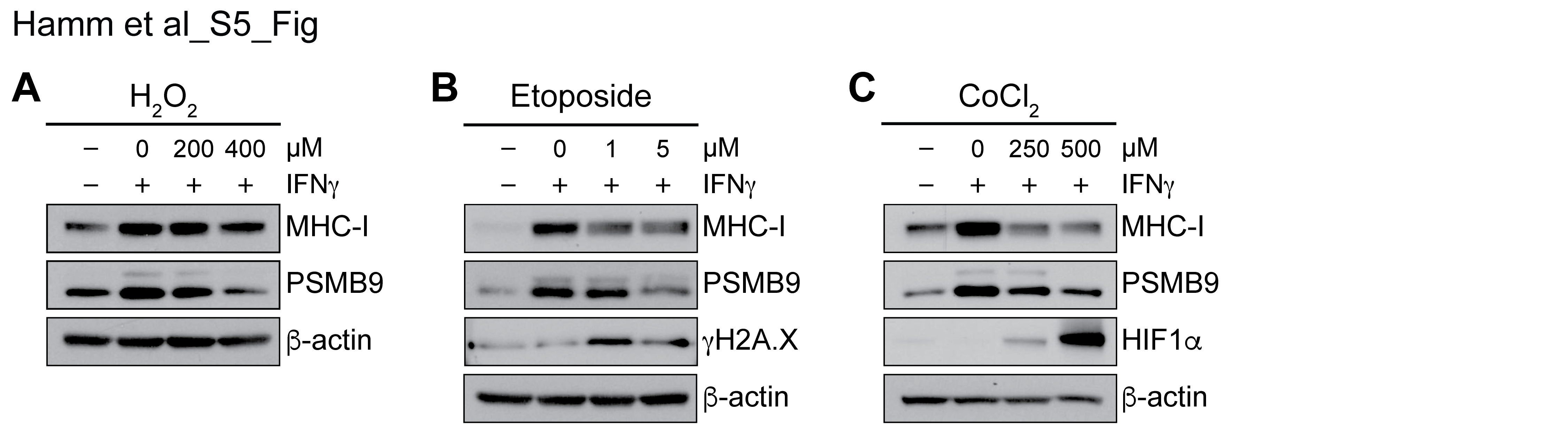

Supplement: S5 Fig — (A) Immunoblot analysis of MB135 myoblasts treated with hydrogen peroxide (H2O2) to induce oxidative stress, (B) etoposide to induce DNA damage, or (C) cobalt chloride (CoCl2) to mimic hypoxia. Cells were treated with stress-inducing reagents for 24 hours, followed by a 16-hour incubation in media resupplemented with cell stress–inducing reagent plus IFNγ. Beta-actin serves as loading control. IFNγ, interferon gamma; MHC-I, major histocompatibility complex class I. (TIF) [file pbio.3002317.s005.tif]

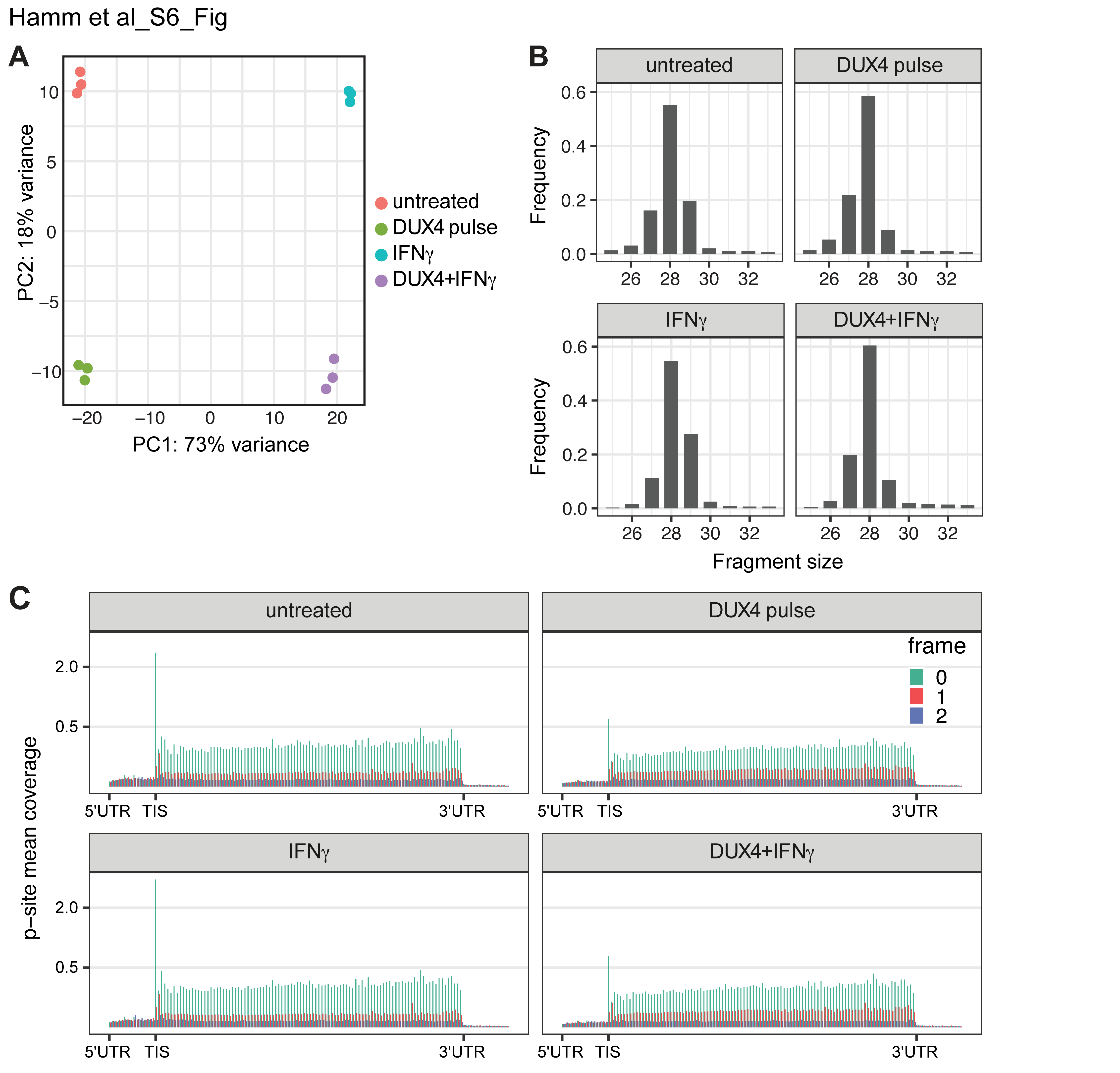

Supplement: S6 Fig — (A) PCA of gene expression in Ribo-seq biological replicates, n = 3; see S1 Data. (B) Length distribution of RPFs; see S1 Data. All downstream analysis was restricted to the dominant fragment size of 26–29 nt. (C) Metagene coverage of P-sites in 3 different reading frames (green, red, blue) over 5′ UTR, TIS, CDS, and 3′ UTR regions; data represent the average of biological replicates for each treatment condition, n = 3. CDS, coding sequence; nt, nucleotide; PCA, principal component analysis; RPF, ribosome-protected fragment; TIS, translation initiation site. (TIF) [file pbio.3002317.s006.tif]

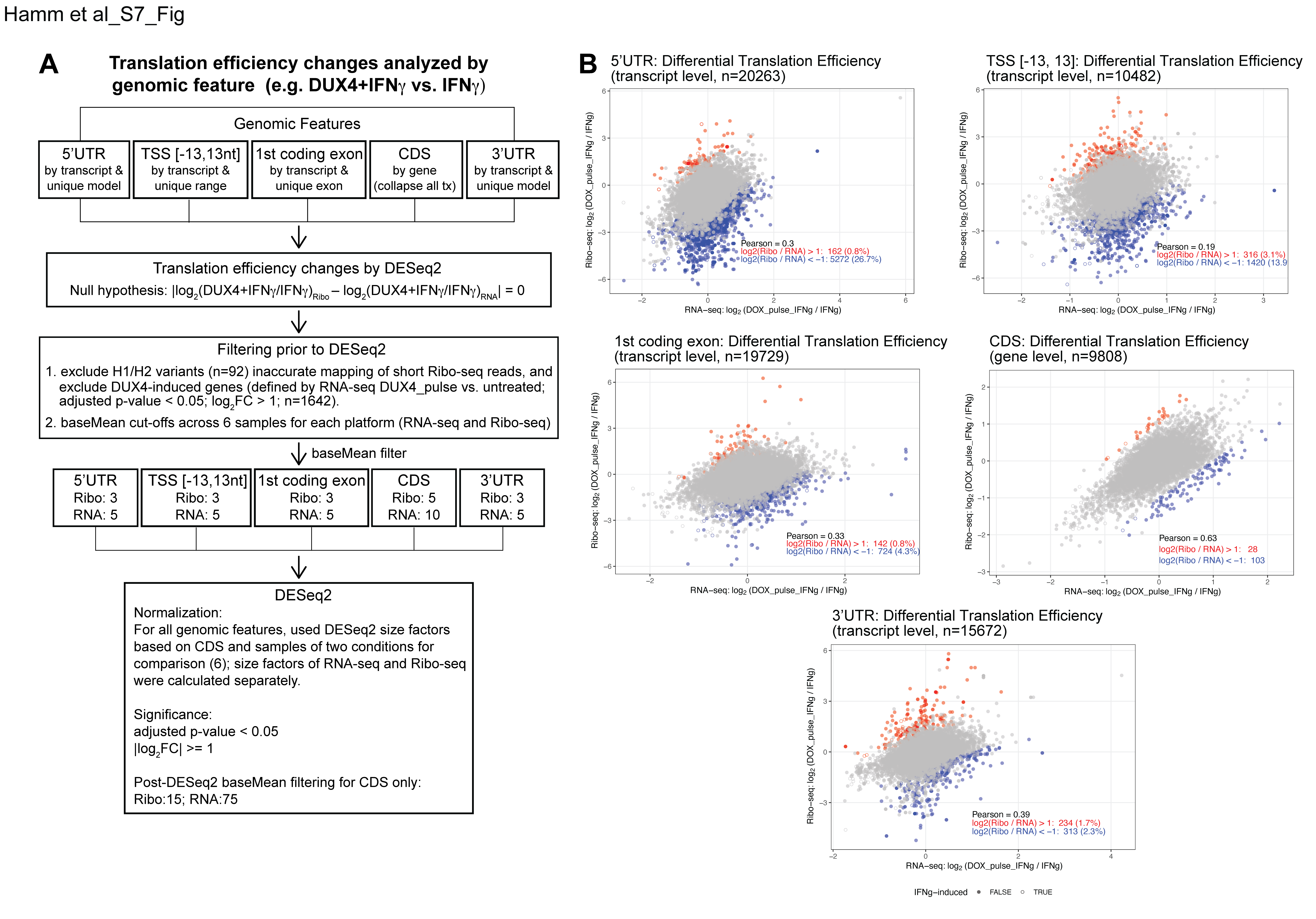

Supplement: S7 Fig — (A) Workflow of differential analysis and filtering prior to DESeq2 analysis to determine translation efficiency at genomic features. (B) Scatter plots of reads aligning to annotated genomic features at the transcript or gene level as indicated. Log2 fold-change values represent the average of biological replicates for DUX4 pulse+IFNγ condition relative to IFNγ treatment alone in MB135iDUX4 myoblasts, n = 3. Translationally up-regulated mRNAs (red) and translationally down-regulated mRNAs (blue) are highlighted (|log2FC>1|, p-adj<0.05, n = 3). DUX4, double homeobox protein 4; IFNγ, interferon gamma; RNA-seq, RNA sequencing. (TIF) [file pbio.3002317.s007.tif]

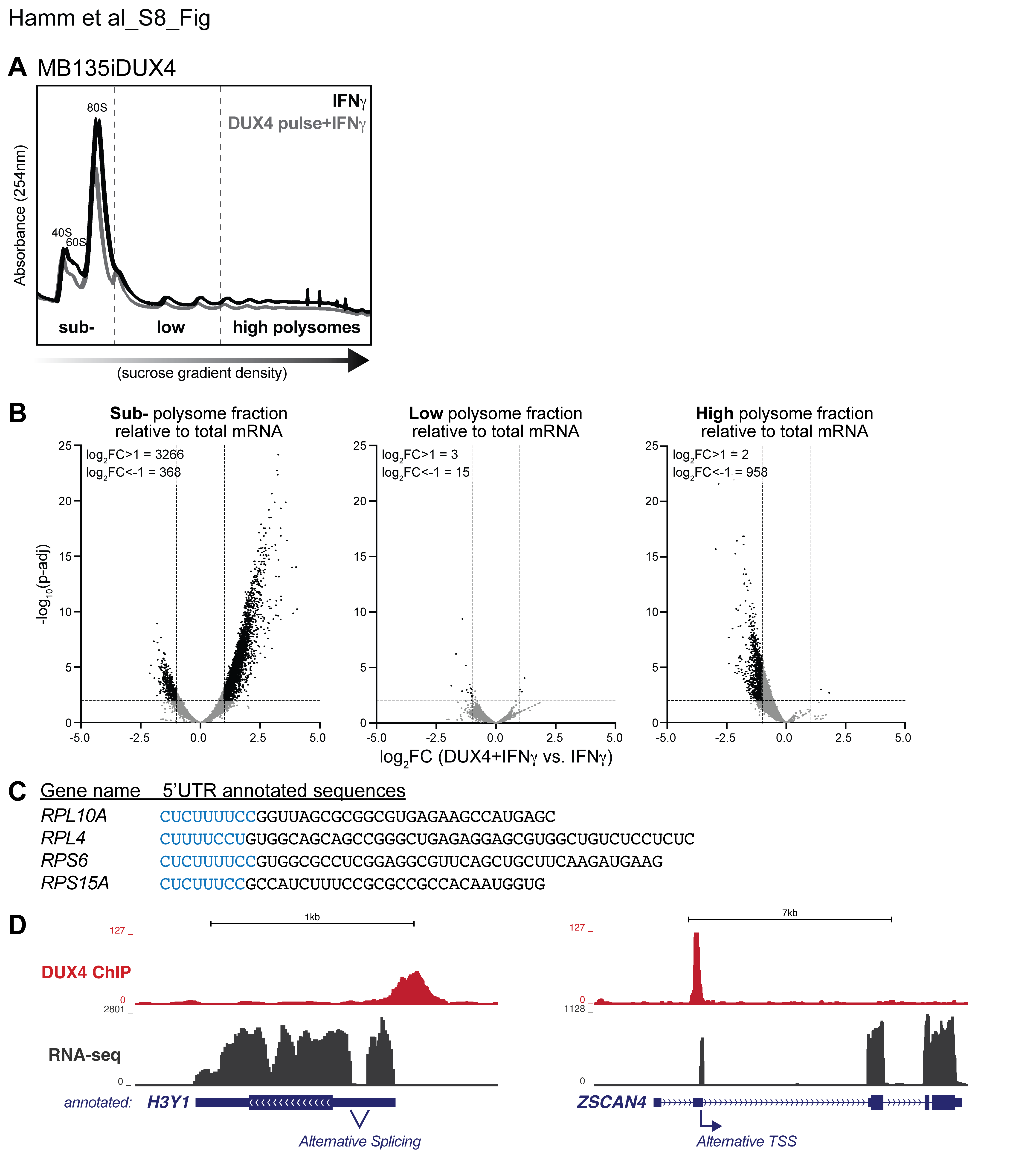

Supplement: S8 Fig — (A) Absorbance at 254 nm across a density gradient fractionation system. Traces represent biological replicates for each treatment condition, n = 3 (black = IFNγ; gray = DUX4 pulse+IFNγ harvested at 68 hours). (B) Differential RNA-seq analysis of sub-, low, and high polysome fractions relative to total mRNA levels. Volcano plot showing log2 fold-change differential abundance in cells treated with DUX4 pulse+IFNγ versus IFNγ (significance defined as basemean>50, |log2FC>1|, p-adj<0.01, n = 3); see S4 Data. (C) 5′ UTR sequences of select TOP mRNAs (blue = TOP motif). (D) Two representative direct DUX4-target genes with alternative 5′ UTRs based on RNA-seq alignment shown relative to the annotated transcripts. DUX4, double homeobox protein 4; IFNγ, interferon gamma; RNA-seq, RNA sequencing; TOP, terminal oligopyrimidine. (TIF) [file pbio.3002317.s008.tif]
