# Supplementary material for: The transcription factor DUX4 orchestrates translational reprogramming by broadly suppressing translation efficiency and promoting expression of DUX4-induced mRNAs
Source: PLoS Biol. 2023 Sep 25;21(9):e3002317. doi: 10.1371/journal.pbio.3002317 (PMC10553841; doi:10.1371/journal.pbio.3002317)

Fig. 1B

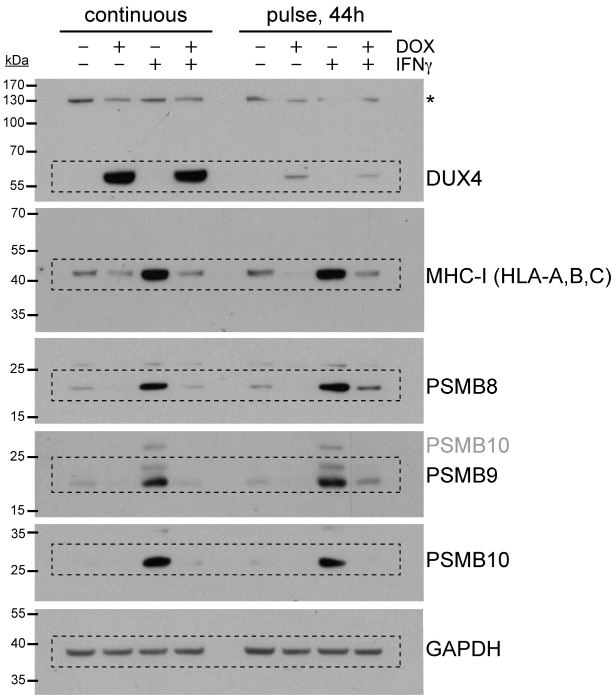

* indicates non-specific immunodetection.

Gray label indicates a blot probed with multiple antibodies for different molecular weight proteins.

Fig. 1D

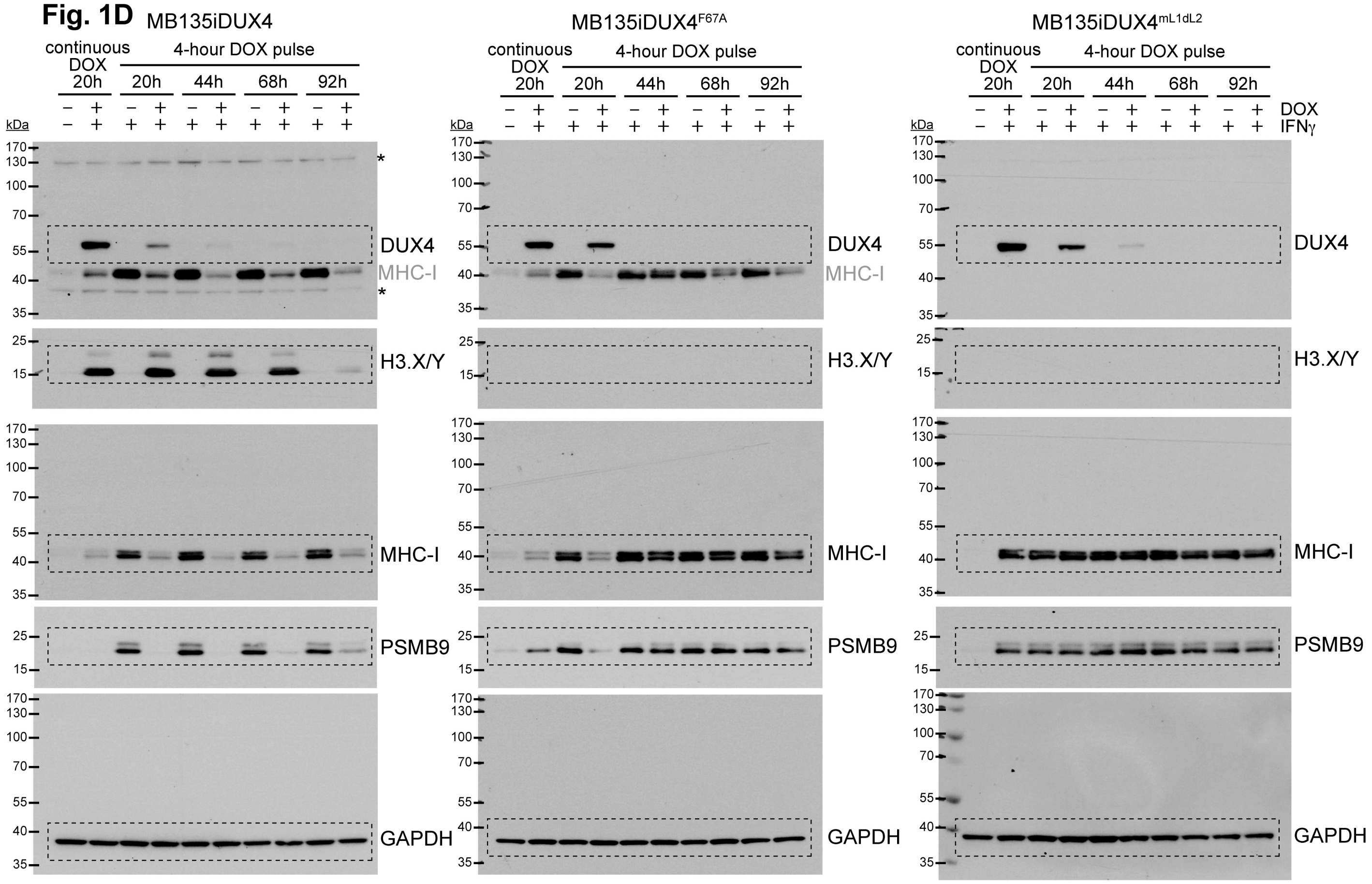

Fig. 2A MB135iDUX4

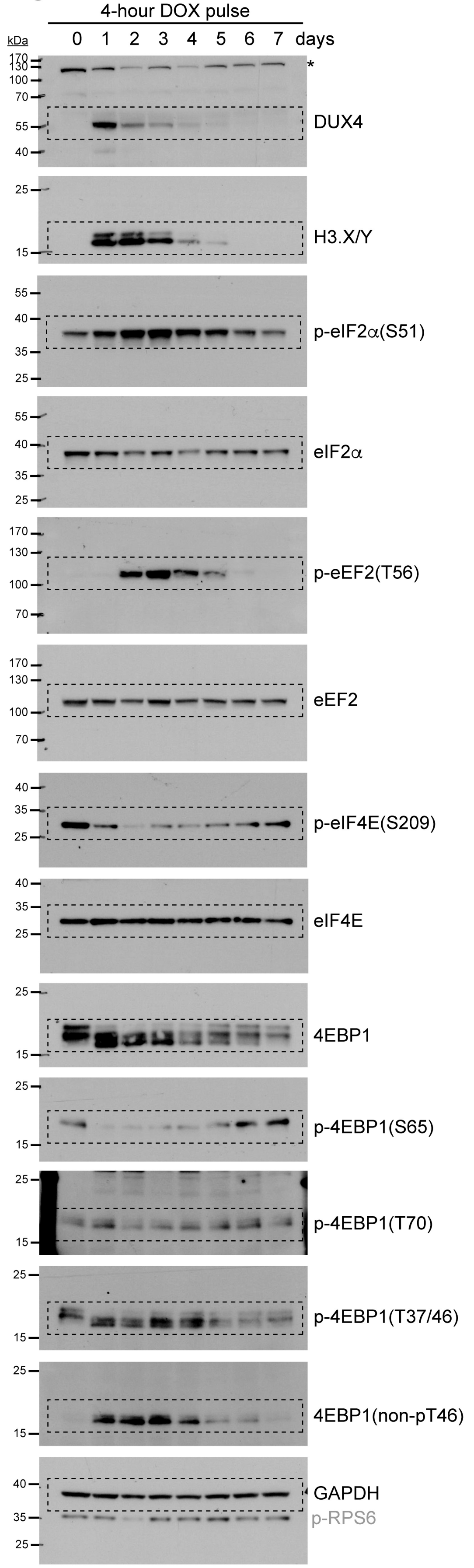

Fig. 2B MB135iDUX4

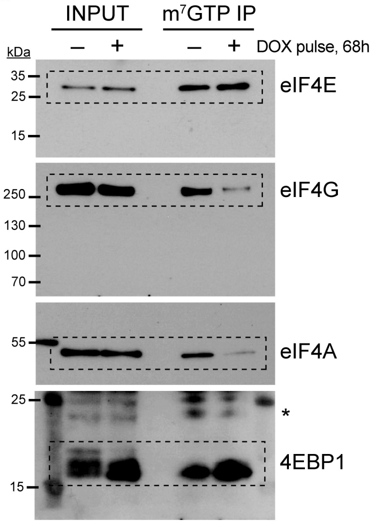

Fig. 2C

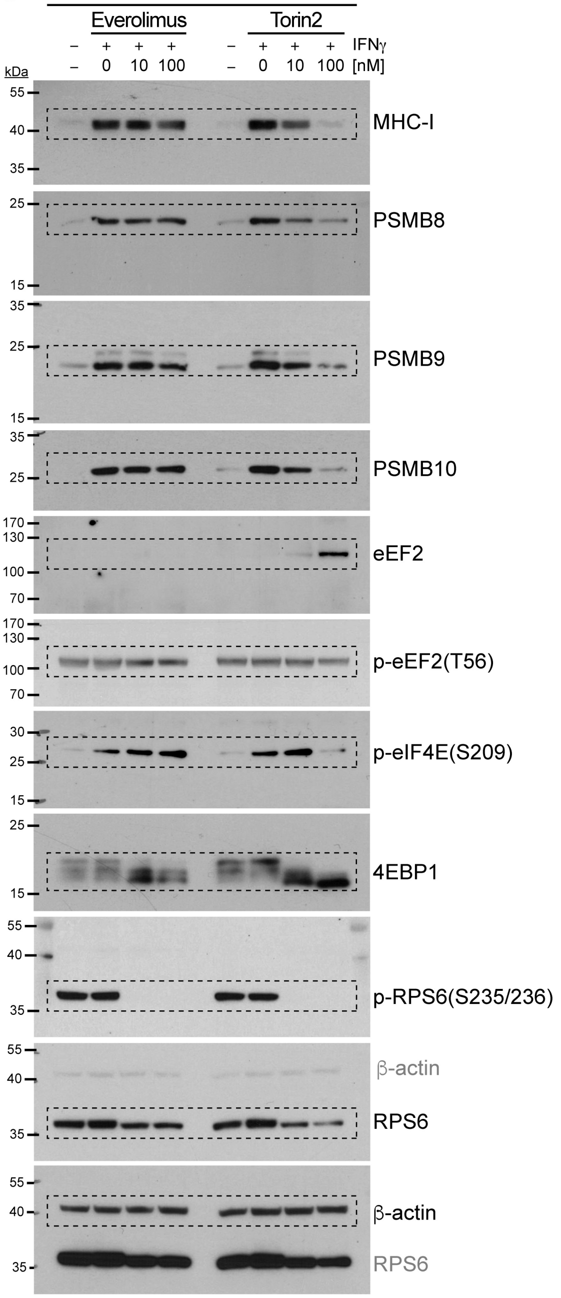

Fig. 3J

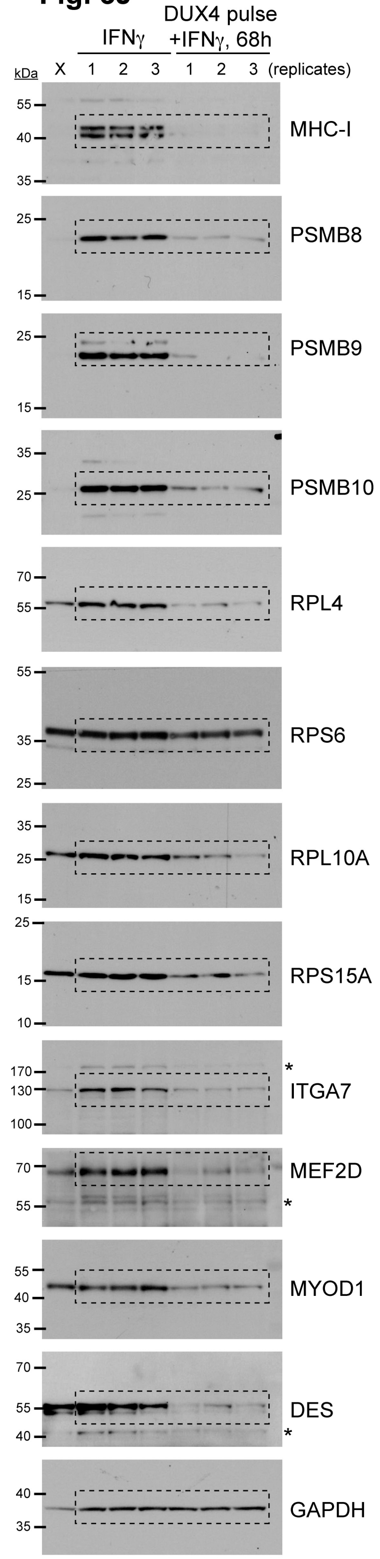

Fig. 4B

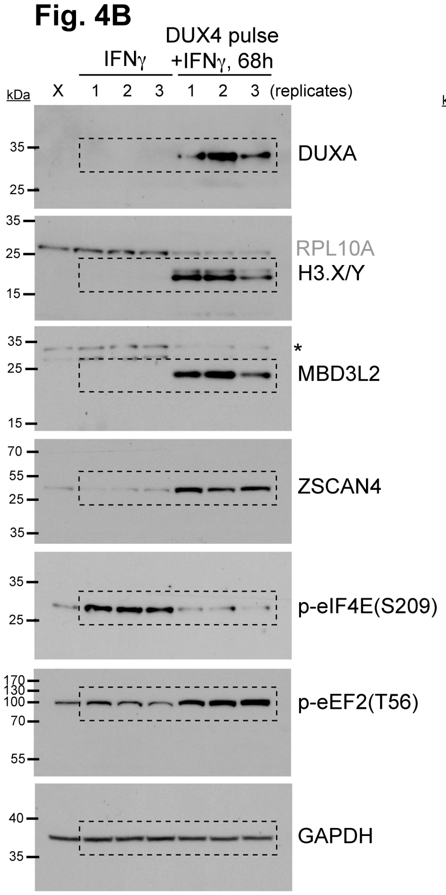

S1A Fig.

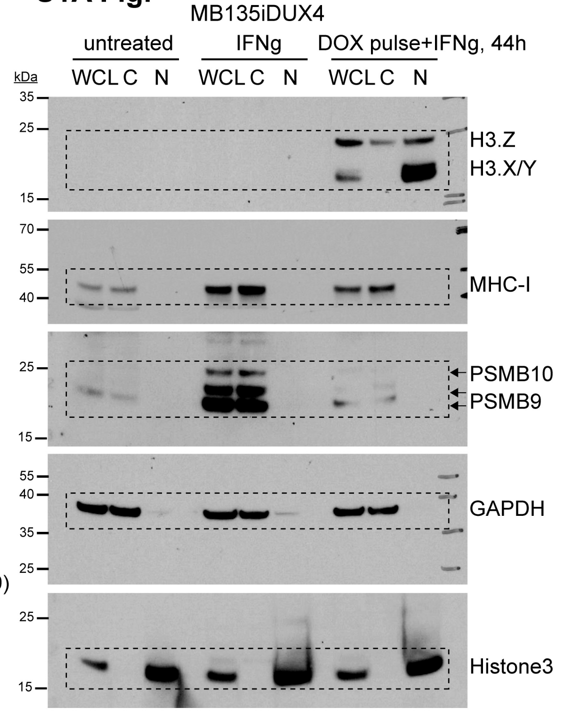

S2A Fig. MB135iDUX4

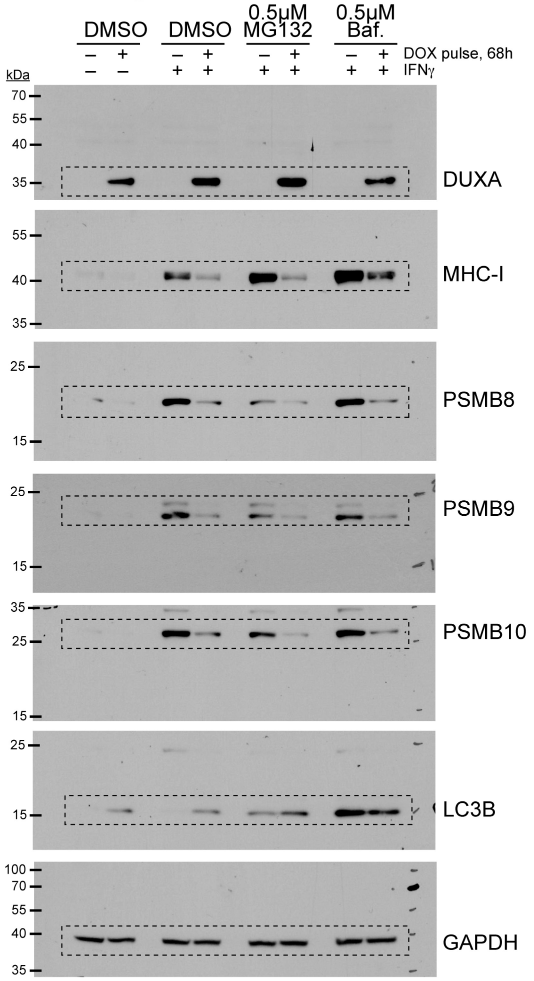

S3A Fig.

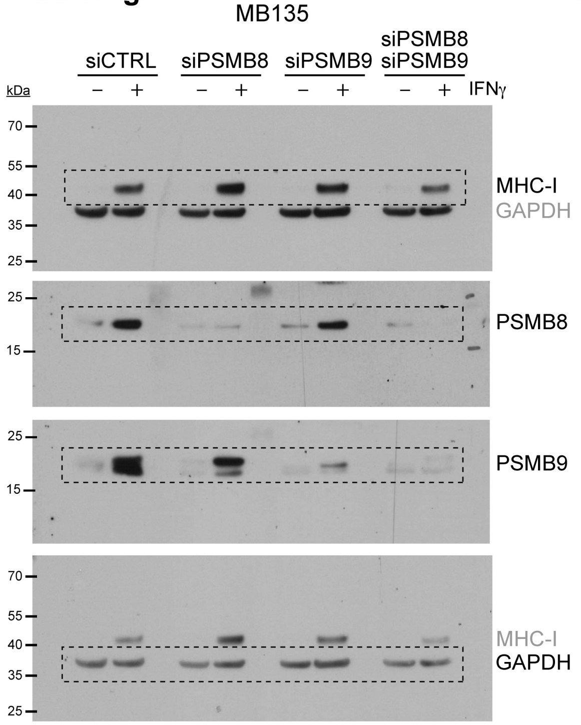

S3B Fig.

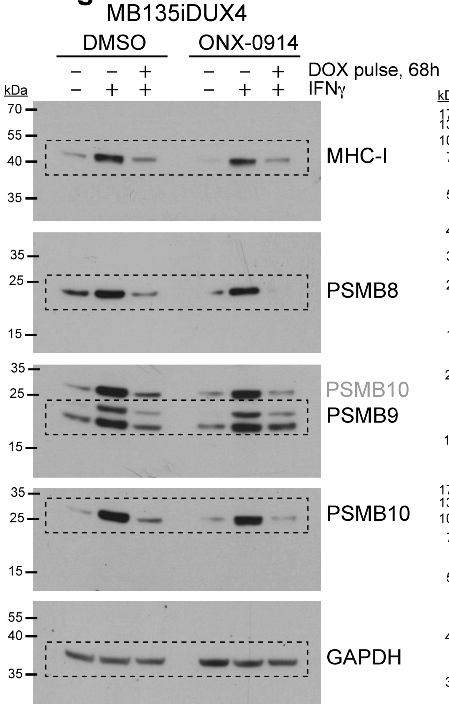

S3D Fig.

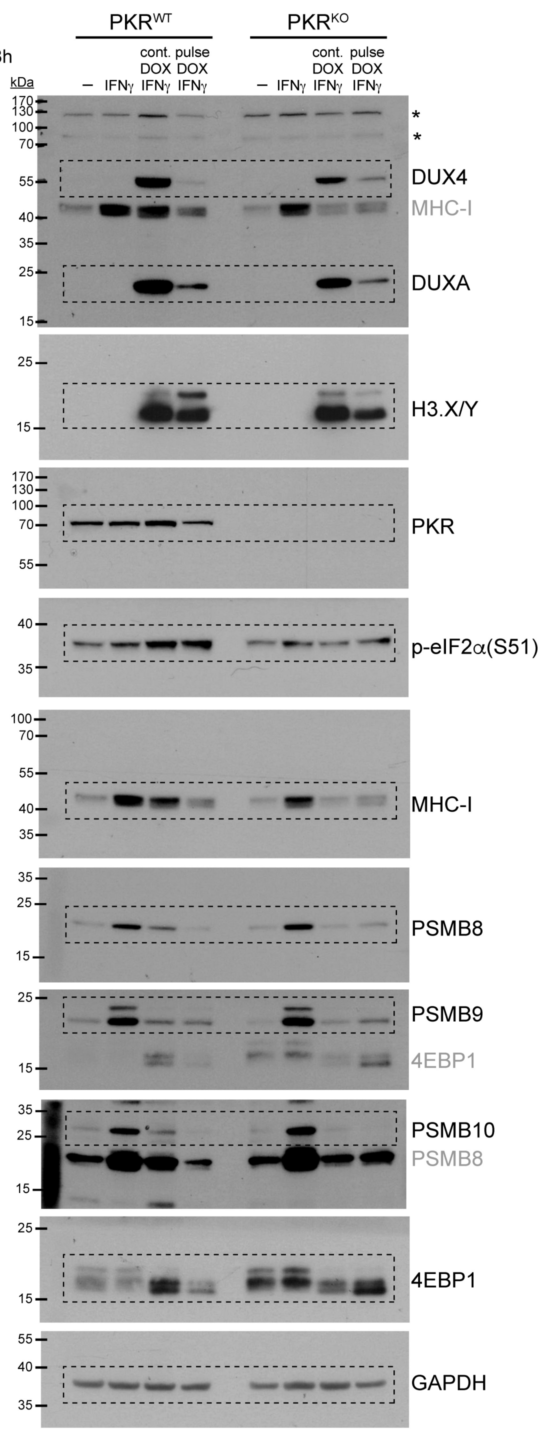

S4C Fig.

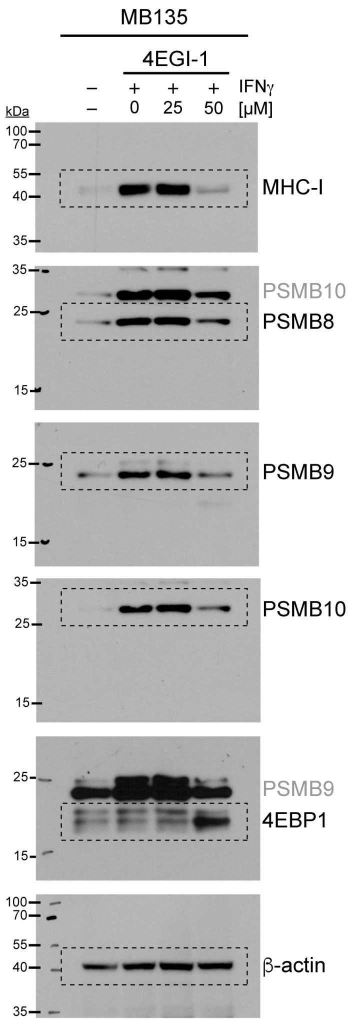

S5A Fig.

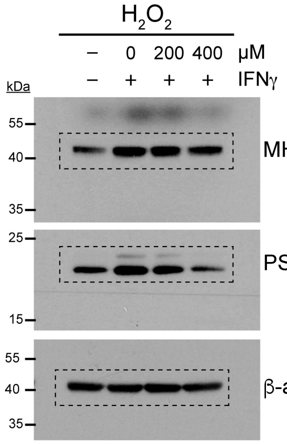

S5B Fig.

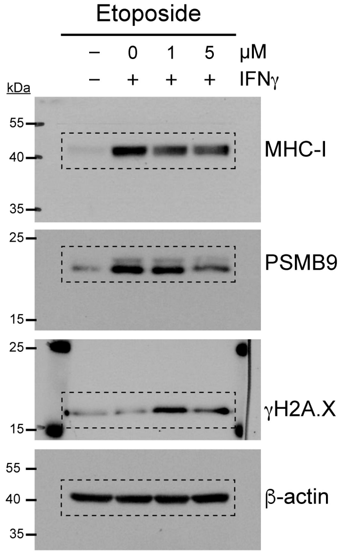

S5C Fig.

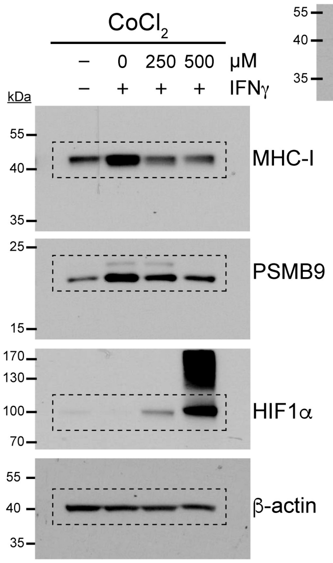

Fig. 2F

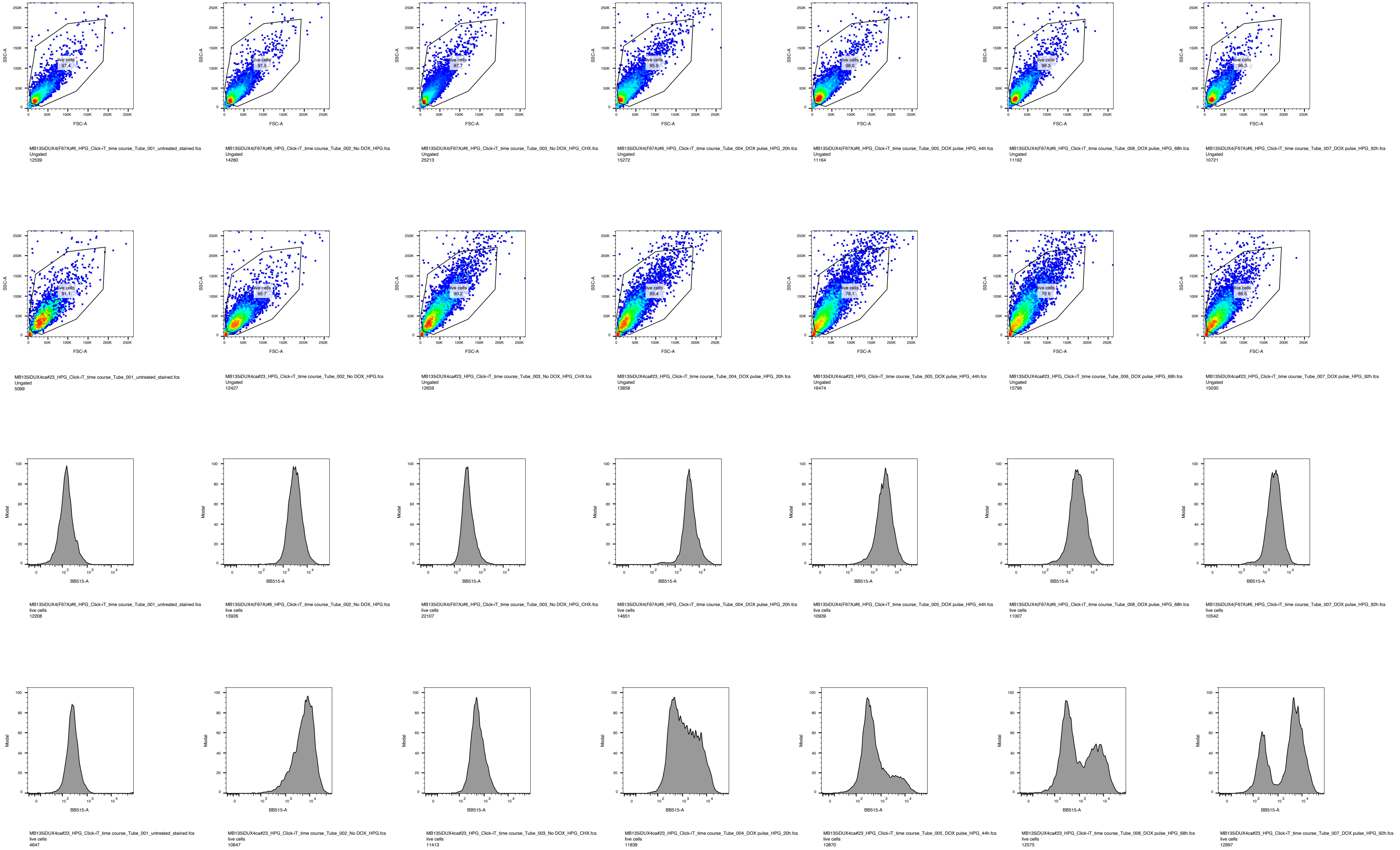
live cells 97.4
live cells 97.5
live cells 87.7
live cells 95.9
live cells 98.0
live cells 98.3
live cells 98.3
FSC-A
SSC-A
MB135iDUX4(F67A)#6_HPG_Click-iT_time course_Tube_001_untreated_stained.fcs Ungated 12539
MB135iDUX4(F67A)#6_HPG_Click-iT_time course_Tube_002_No DOX_HPG.fcs Ungated 14280
MB135iDUX4(F67A)#6_HPG_Click-iT_time course_Tube_003_No DOX_HPG_CHX.fcs Ungated 25213
MB135iDUX4(F67A)#6_HPG_Click-iT_time course_Tube_004_DOX pulse_HPG_20h.fcs Ungated 15272
MB135iDUX4(F67A)#6_HPG_Click-iT_time course_Tube_005_DOX pulse_HPG_44h.fcs Ungated 11164
MB135iDUX4(F67A)#6_HPG_Click-iT_time course_Tube_006_DOX pulse_HPG_68h.fcs Ungated 11192
MB135iDUX4(F67A)#6_HPG_Click-iT_time course_Tube_007_DOX pulse_HPG_92h.fcs Ungated 10721
live cells 91.1
live cells 85.7
live cells 90.2
live cells 85.4
live cells 78.1
live cells 79.6
live cells 86.5
MB135iDUX4ca#23_HPG_Click-iT_time course_Tube_001_untreated_stained.fcs Ungated 5099
MB135iDUX4ca#23_HPG_Click-iT_time course_Tube_002_No DOX_HPG.fcs Ungated 12427
MB135iDUX4ca#23_HPG_Click-iT_time course_Tube_003_No DOX_HPG_CHX.fcs Ungated 12659
MB135iDUX4ca#23_HPG_Click-iT_time course_Tube_004_DOX pulse_HPG_20h.fcs Ungated 13858
MB135iDUX4ca#23_HPG_Click-iT_time course_Tube_005_DOX pulse_HPG_44h.fcs Ungated 16474
MB135iDUX4ca#23_HPG_Click-iT_time course_Tube_006_DOX pulse_HPG_68h.fcs Ungated 15796
MB135iDUX4ca#23_HPG_Click-iT_time course_Tube_007_DOX pulse_HPG_92h.fcs Ungated 15030
Modal
BB515-A
MB135iDUX4(F67A)#6_HPG_Click-iT_time course_Tube_001_untreated_stained.fcs live cells 12208
MB135iDUX4(F67A)#6_HPG_Click-iT_time course_Tube_002_No DOX_HPG.fcs live cells 13926
MB135iDUX4(F67A)#6_HPG_Click-iT_time course_Tube_003_No DOX_HPG_CHX.fcs live cells 22107
MB135iDUX4(F67A)#6_HPG_Click-iT_time course_Tube_004_DOX pulse_HPG_20h.fcs live cells 14651
MB135iDUX4(F67A)#6_HPG_Click-iT_time course_Tube_005_DOX pulse_HPG_44h.fcs live cells 10939
MB135iDUX4(F67A)#6_HPG_Click-iT_time course_Tube_006_DOX pulse_HPG_68h.fcs live cells 11007
MB135iDUX4(F67A)#6_HPG_Click-iT_time course_Tube_007_DOX pulse_HPG_92h.fcs live cells 10542
MB135iDUX4ca#23_HPG_Click-iT_time course_Tube_001_untreated_stained.fcs live cells 4647
MB135iDUX4ca#23_HPG_Click-iT_time course_Tube_002_No DOX_HPG.fcs live cells 10647
MB135iDUX4ca#23_HPG_Click-iT_time course_Tube_003_No DOX_HPG_CHX.fcs live cells 11413
MB135iDUX4ca#23_HPG_Click-iT_time course_Tube_004_DOX pulse_HPG_20h.fcs live cells 11839
MB135iDUX4ca#23_HPG_Click-iT_time course_Tube_005_DOX pulse_HPG_44h.fcs live cells 12870
MB135iDUX4ca#23_HPG_Click-iT_time course_Tube_006_DOX pulse_HPG_68h.fcs live cells 12575
MB135iDUX4ca#23_HPG_Click-iT_time course_Tube_007_DOX pulse_HPG_92h.fcs live cells 12997

Fig. 4G

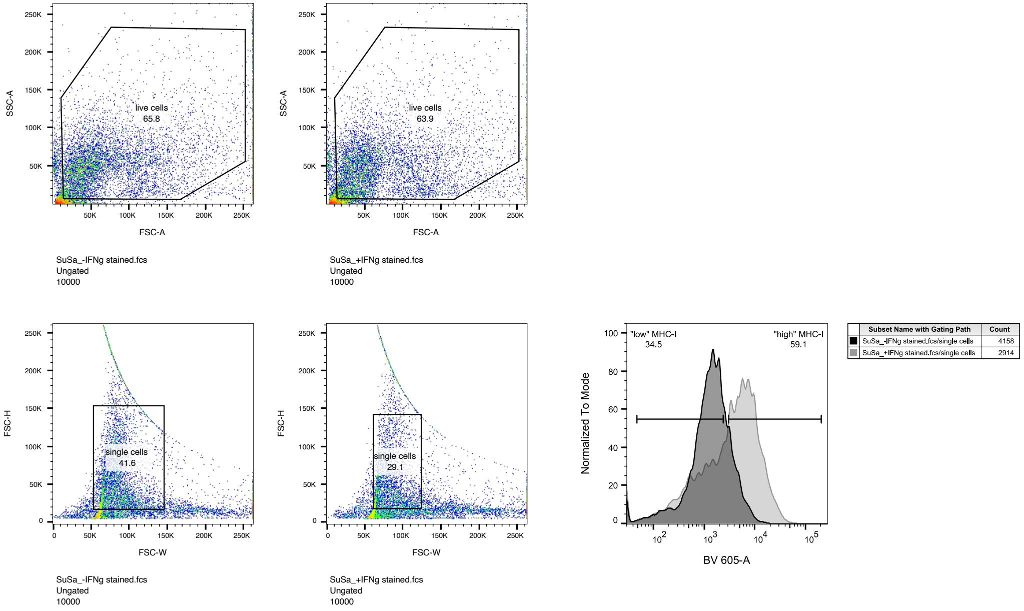

Fig. 4I-J

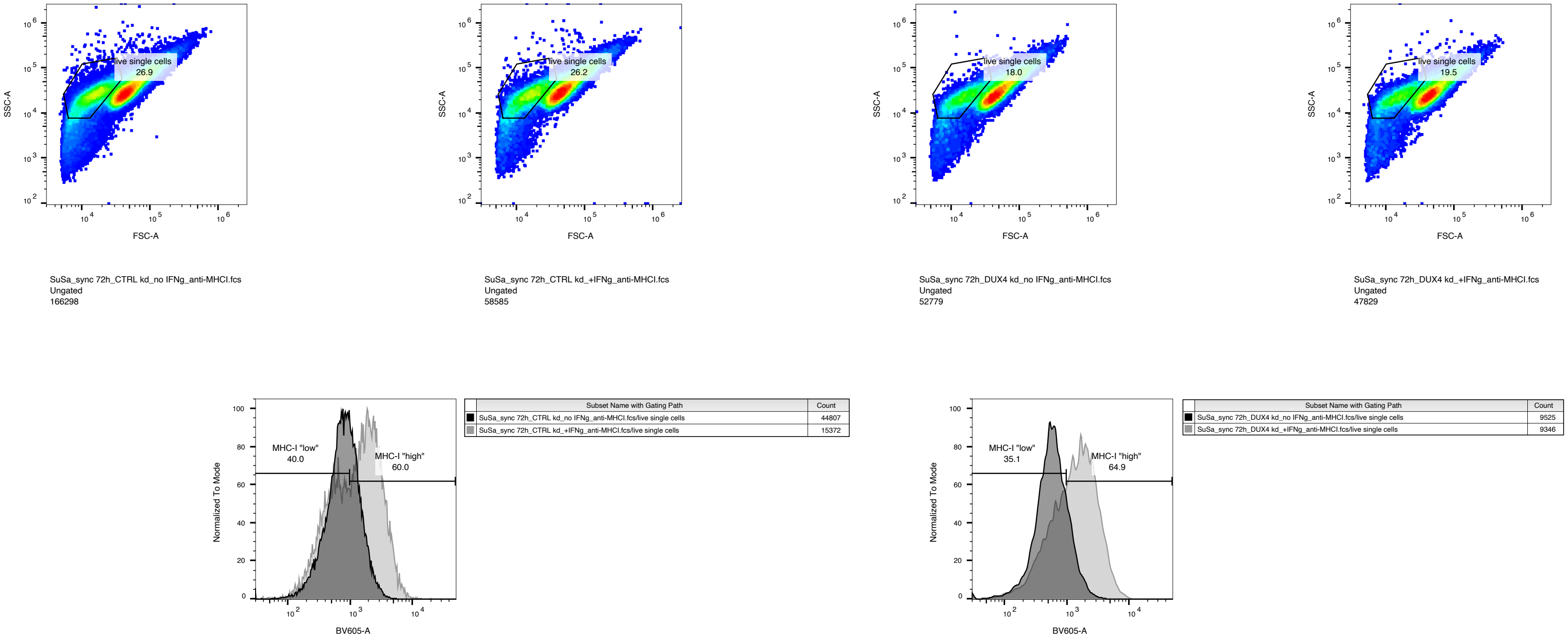

Supplement: S1 Raw Images — (PDF) [file pbio.3002317.s014.pdf]
